# Enantioselective Synthesis of the Sex Pheromone of *Sitodiplosis mosellana* (Géhin) and Its Stereoisomers

**DOI:** 10.3390/molecules30030671

**Published:** 2025-02-03

**Authors:** Jianan Wang, Xiaoyang Li, Yun Zhou, Qinghua Bian, Jiangchun Zhong

**Affiliations:** 1 Department of Applied Chemistry, China Agricultural University, Beijing 100193, Chinabianqinghua@cau.edu.cn (Q.B.); 2 Institute of Industrial Crops, Shandong Academy of Agricultural Sciences, Jinan 250100, China; zysass2021@163.com

**Keywords:** orange wheat blossom midge, chiral source, sex pheromone, asymmetric synthesis

## Abstract

(2*S*,7*S*)-2,7-Nonanediyl dibutyrate is the sex pheromone of *Sitodiplosis mosellana* (Géhin). In this study, this sex pheromone and its three stereoisomers were prepared. Central to this strategy was the ring opening of chiral epoxide with an alkynyllithium and the hydrogenation of the triple bond. Moreover, this approach consisted of six steps, and the total yields were 59–64%.

## 1. Introduction

The orange wheat blossom midge, *Sitodiplosis mosellana* (Géhin) (Diptera: Cecidomyiidae), is a notorious pest worldwide [1,2], which has caused significant yield losses in wheat (*Triticum aestivum* Linnaeus) [3,4]. The adult females often oviposit on wheat heads, and the larvae feed on the developing seeds [5]. This leads to shriveling and presprouting damage, resulting in a reduction in both the yield and quality of wheat harvested [6,7]. The current integrated pest management programs for *S. mosellana* mainly rely on pesticides [8,9], the conservation of natural enemies [10] and resistant cultivars [11,12].

**Figure 1:**
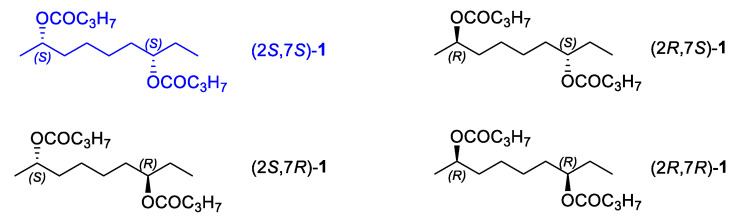
Sex pheromone of *S. mosellana* and its stereoisomers.

Pheromone-based pest control is an alternative strategy with the advantages of environmental friendliness, high efficiency and rarely inducing pest resistance [13,14]. In 2000, Gries identified that the sex pheromone of *S. mosellana* was (2*S*,7*S*)-2,7-nonanediyl dibutyrate ((2*S*,7*S*)-1) (Figure 1) via coupled gas chromatographic-electroantennographic detection (GC-EAD) with GC–mass spectrometry (MS) and trap experiments in wheat fields [15]. Furthermore, Gries prepared (2*S*,7*S*)-**1** using CuI, which catalyzed the reaction of *S*-propylene oxide with Grignard reagent, and through the hydrolytic kinetic resolution of epoxide using Jacobsen’s catalyst [15]. The pheromone could be utilized for monitoring and trapping the orange wheat blossom midge [16,17], which has attracted significant interest from chemists. Previous syntheses have employed various approaches, including chiral sources of (*S*)-but-3-yn-2-ol [18,19] and (*S*)-5-hexen-2-ol [20], and the esterification of racemic nonanediyl dibutyrate using *Pseudomonas cepacia* Burkh lipase [21]. To further research the biological activities of the sex pheromone, herein, new and efficient synthesis of the sex pheromone of *S. mosellana* and its stereoisomers (Figure 1) is achieved.

## 2. Results and Discussion

### 2.1. Retrosynthetic Analysis

**Scheme 1:**
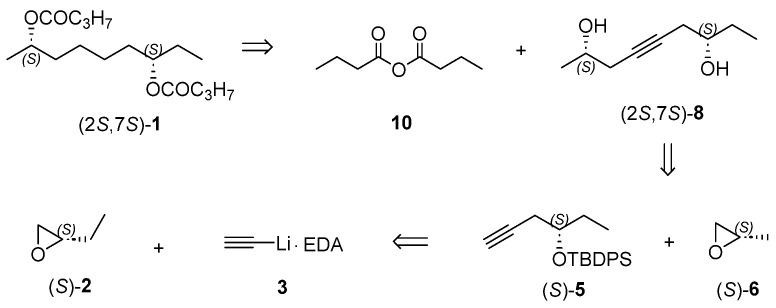
Retrosynthetic analysis of sex pheromone of *S. mosellana* (2*S*,7*S*)-**1**.

The retrosynthetic analysis of the sex pheromone of *S. mosellana* (2*S*,7*S*)-**1** is shown in Scheme 1. The target pheromone (2*S*,7*S*)-**1** was obtained through the acylation of alkynyl diol (2*S*,7*S*)-**8** with butyric anhydride (**10**). The chiral secondary hydroxy at C2 of (2*S*,7*S*)-**8** was envisioned to be constructed through the addition of (*S*)-2-methyloxirane ((*S*)-**6**) with chiral TBDPS ether (*S*)-**5** and *n*-BuLi. The other stereocenter of (2*S*,7*S*)-**8** was obtained from the chiral source (*S*)-2-ethyloxirane ((*S*)-**2**). Following a similar procedure for sex pheromone (2*S*,7*S*)-**1**, its stereoisomers (2*R*,7*S*)-**1**, (2*S*,7*R*)-**1**, (2*R*,7*R*)-**1** were prepared.

### 2.2. Synthesis of Chiral TBDPS Ethers

**Scheme 2:**
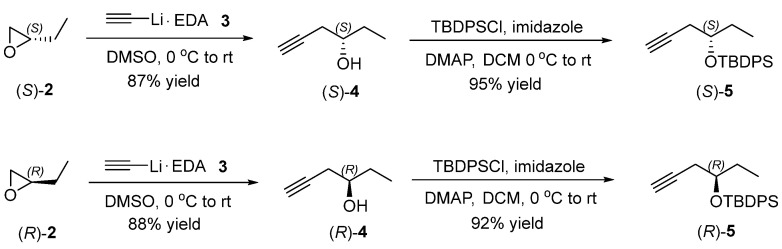
Synthesis of chiral TBDPS ethers (*S*)- and (*R*)-**5**.

Based on the retrosynthetic analysis of the sex pheromone (2*S*,7*S*)-**1**, our synthesis started from the preparation of chiral TBDPS ethers (*S*)- and (*R*)-**5** (Scheme 2). The reaction of (*S*)-2-ethyloxirane ((*S*)-**2**) with lithium acetylide ethylenediamine complex (**3**) in DMSO yielded (*S*)-hex-5-yn-3-ol ((*S*)-**4**) (87% yield) [22,23]. The specific rotation of chiral alcohol (*S*)-**4** was identical to that in the literature data [24]. The secondary hydroxy of (*S*)-**4** was then protected by a treatment with TBDPSCl and imidazole to provide chiral TBDPS ether (*S*)-**5** in a 95% yield [25,26]. Similarly, (*R*)-tert-butyl(hex-5-yn-3-yloxy)diphenylsilane ((*R*)-**5**) was prepared via the ring opening of (*R*)-2-ethyloxirane ((*R*)-**2**) with lithium acetylide ethylenediamine complex (**3**) and the protection of hydroxy of (*R*)-**4** with TBDPSCl.

### 2.3. Synthesis of Sex Pheromone of S. mosellana 

**Scheme 3:**
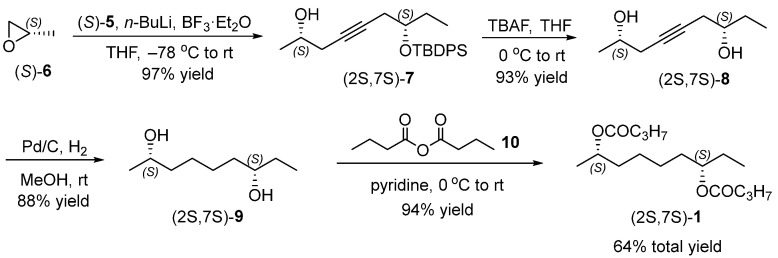
Synthesis of sex pheromone of *S. mosellana*.

With the key chiral block (*S*)- **5** in hand, we next prepared the sex pheromone of *S. mosellana* (Scheme 3). In the presence of boron trifluoride-diethyl etherate, the addition of (*S*)-2-methyloxirane ((*S*)-**6**) with alkynyllithium, prepared in situ from the chiral TBDPS ether (*S*)-**5** and *n*-BuLi, provided (2*S*,7*S*)-7-((tert-butyldiphenylsilyl)oxy)non-4-yn-2-ol ((2*S*,7*S*)-**7**) almost quantitatively [27,28]. The subsequent deprotection with TBAF gave (2*S*,7*S*)-non-4-yne-2,7-diol ((2*S*,7*S*)-**8**) in a 93% yield [29]. Finaly, the hydrogenation of the triple bond of chiral alkynyl diol (2*S*,7*S*)-**8** resulted in the formation of (2*S*,7*S*)-nonane-2,7-diol ((2*S*,7*S*)-**9**) [30,31], which was treated with butyric anhydride (**10**) to yield (2*S*,7*S*)-nonane-2,7-diyl dibutyrate ((2*S*,7*S*)-**1**) [32,33]. The NMR spectra, HRMS, and specific rotation of the sex pheromone (2*S*,7*S*)-**1** matched those in reference [20].

### 2.4. Synthesis of Stereoisomers of S. mosellana Sex Pheromone

**Scheme 4:**
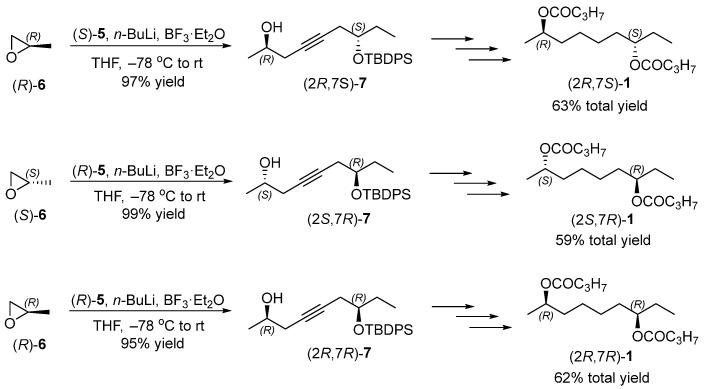
Synthesis of stereoisomers of *S. mosellana* sex pheromone.

Having achieved the synthesis of the sex pheromone of *S. mosellana*, we next investigated the preparation of its stereoisomers (Scheme 4). According to a similar procedure for the sex pheromone (2*S*,7*S*)-**1**, its stereoisomers (2*R*,7*S*)-**1**, (2*S*,7*R*)-**1** and (2*R*,7*R*)-**1** were synthesized from chiral TBDPS ether (*S*)-**5** and (*R*)-2-methyloxirane ((*R*)-**6**); chiral TBDPS ether (*R*)-**5** and (*S*)-2-methyloxirane ((*S*)-**6**); and chiral TBDPS ether (*R*)-**5** and (*R*)-2-methyloxirane ((*R*)-**6**), respectively. The structures of these stereoisomers were characterized by specific rotation, NMR spectra and HRMS (The NMR spectra are available in the Supplementary Materials).

## 3. Materials and Methods

### 3.1. General Information

Unless otherwise stated, all reactions were performed under an argon atmosphere with a Schlenk line system. All commercial reagents and starting materials were used as received. The chiral reagents of (*S*)-2-ethyloxirane (268 RMB/100 mL), (*R*)-2-ethyloxirane (268 RMB/100 mL), (*S*)-2-methyloxirane (104 RMB/5 g) and (*R*)-2-methyloxirane (240 RMB/5 g) were purchased from Anhui Senrise Technology Co., Ltd., (Anhui, China). Dichloromethane, tetrahydrofuran, methanol and dimethyl sulfone were purchased from Beijing Ouhe Technology Co., Ltd., (Beijing, China), and purified according to standard procedures. ^1^H NMR and ^13^C NMR spectra were recorded on a Bruker Ascend^TM^ 500 MHz spectrometer (Bruker Corporation, Billerica, MA, USA) at 500 MHz and 125 MHz, respectively. The chemical shifts were reported in ppm using TMS (0.00 ppm) and CDCl_3_ (77.16 ppm) as internal standards. High-resolution mass spectra (HRMS) were obtained on a Waters LCT Premier^TM^ (Waters Corporation, Milford, MA, USA) with an electrospray ionization (ESI) mass spectrometer. Optical rotations were measured on a Rudolph AUTOPOL-IV polarimeter (Rudolph Research Analytical, Flanders, NJ, USA).

### 3.2. Synthesis of (S)-5-hexyn-3-ol ((S)-***4***)

Under an argon atmosphere, (*S*)-2-ethyloxirane ((*S*)-**2**) (5.00 g, 69.35 mmol, >99% ee, 1.0 equiv.) was added slowly to a stirred suspension of lithium acetylide ethylenediamine complex (**3**) (15.96 g, 173.38 mmol, 2.5 equiv.) in DMSO (100 mL) at 0 °C. The reaction mixture was allowed to warm to room temperature and stirred continuously overnight. The reaction was then quenched by adding cold water (50 mL), followed by extraction with DCM (3 × 30 mL). The combined organic layers were washed with brine (30 mL), dried over anhydrous Na_2_SO_4_ and concentrated under reduced pressure to yield the crude product. The crude product was purified by silica gel column chromatography using an eluent of ethyl acetate and petroleum ether (1:5) to produce (*S*)-hex-5-yn-3-ol ((*S*)-**4**) (5.92 g, 87% yield) as a colorless oil. [*α*]_D_^25^ = +2.78 (c 1.29, CHCl_3_). Lit. [24] [*α*]_D_ = +3.00 (c 2.00, CHCl_3_). ^1^H NMR (500 MHz, CDCl_3_) δ 3.65–3.60 (m, 1H), 2.39–2.34 (m, 1H), 2.28–2.23 (m, 1H), 2.04 (br s, 1H), 1.98 (t, *J* = 2.7 Hz, 1H), 1.57–1.45 (m, 2H), 0.89 (t, *J* = 7.5 Hz, 3H). ^13^C NMR (125 MHz, CDCl_3_) δ 81.0, 71.3, 70.8, 29.2, 27.0, 10.0. HRMS (ESI, *m*/*z*): calculated for [M + Na]^+^ C_6_H_10_ONa 121.0624, found: 121.0615.

### 3.3. Synthesis of (R)-5-hexyn-3-ol ((R)-***4***)

Using a similar procedure for chiral alcohol (*S*)-**4**, the addition of lithium acetylide ethylenediamine complex **3** (15.96 g, 173.38 mmol, 2.5 equiv.) to (*R*)-2-ethyloxirane ((*R*)-**2**) (5.00 g, 69.35 mmol, >99% ee, 1.0 equiv.) yielded (*R*)-5-Hexyn-3-ol ((*R*)-**4**) (5.98 g, 88% yield) as a colorless oil. [*α*]_D_^25^ = −4.45 (c 0.99, CHCl_3_). Lit. [24] [*α*]_D_ = −4.00 (c 2.00, CHCl_3_). ^1^H NMR (500 MHz, CDCl_3_) δ 3.65–3.60 (m, 1H), 2.39–2.34 (m, 1H), 2.28–2.23 (m, 1H), 1.99–1.98 (m, 1H), 1.90–1.88 (m, 1H), 1.55–1.47 (m, 2H), 0.90 (t, *J* = 7.5 Hz, 3H). ^13^C NMR (125 MHz, CDCl_3_) δ 81.0, 71.3, 70.8, 29.2, 27.0, 10.0. HRMS (ESI, *m/z*): calculated for [M + Na]^+^ C_6_H_10_ONa 121.0624, found: 121.0612.

### 3.4. Synthesis of (S)-tert-butyl(hex-5-yn-3-yloxy)diphenylsilane ((S)-***5***)

Under an argon atmosphere, (*S*)-5-hexyn-3-ol ((*S*)-**4**) (8.54 g, 87.00 mmol, 1.0 equiv.) was added slowly to a solution of DMAP (2.13 g, 17.40 mmol, 0.2 equiv.) and imidazole (14.81 g, 217.50 mmol, 2.5 equiv.) in DCM (200 mL) at 0 °C. Subsequently, TBDPSCl (29.12 g, 104.4 mmol, 1.2 equiv.) was added. The mixture was allowed to warm to room temperature and stirred continuously overnight. The reaction was then quenched by adding water (50 mL), followed by a separation of the lower organic phase. The higher aqueous phase was extracted with DCM (3 × 30 mL). The combined organic layers were washed with brine (30 mL), dried over anhydrous Na_2_SO_4_ and concentrated under reduced pressure to yield the crude product. The crude product was purified by silica gel column chromatography using an eluent of ethyl acetate and petroleum ether (1:20) to produce (*S*)-tert-butyl(hex-5-yn-3-yloxy)diphenylsilane ((*S*)-**5**) (27.8 g, 95% yield) as a light yellow oil. [*α*]_D_^25^ = −26.58 (c 4.91, CHCl_3_). ^1^H NMR (500 MHz, CDCl_3_) δ 7.61–7.58 (m, 4H), 7.30–7.23 (m, 6H), 3.74–3.70 (m, 1H), 2.24–2.14 (m, 2H), 1.76 (t, *J* = 2.7 Hz, 1H), 1.60–1.47 (m, 2H), 0.97 (s, 9H), 0.74 (t, *J* = 7.5 Hz, 3H). ^13^C NMR (125 MHz, CDCl_3_) δ 136.03, 136.00, 134.4, 134.2, 129.8, 127.76, 127.69, 81.4, 72.6, 70.1, 28.7, 27.1, 26.0, 19.5, 9.1. HRMS (ESI, *m*/*z*): calculated for [M + H]^+^ C_22_H_29_OSi 337.1982, found: 339.1954.

### 3.5. Synthesis of (R)-tert-butyl(hex-5-yn-3-yloxy)diphenylsilane ((R)-***5***)

Using a similar procedure for chiral TBDPS ether (*S*)-**5**, the protection of (*R*)-5-hexyn-3-ol ((*R*)-**4**) (8.54 g, 87.00 mmol, 1.0 equiv.) with TBDPSCl (29.12 g, 104.40 mmol, 1.2 equiv.) yielded (*R*)-tert-butyl(hex-5-yn-3-yloxy)diphenylsilane ((*R*)-**5**) (26.9 g, 92% yield) as a light yellow oil. [*α*]_D_^25^ = +25.77 (c 10.58, CHCl_3_). ^1^H NMR (500 MHz, CDCl_3_) δ 7.62–7.58 (m, 4H), 7.33–7.25 (m, 6H), 3.75–3.71 (m, 1H), 2.24–2.15 (m, 2H), 1.79 (t, *J* = 2.7 Hz, 1H), 1.58–1.50 (m, 2H), 0.98 (s, 9H), 0.76 (t, *J* = 7.5 Hz, 3H). ^13^C NMR (125 MHz, CDCl_3_) δ 136.04, 136.01, 134.4, 134.2, 129.8, 127.69, 127.66, 127.64, 81.5, 72.6, 70.0, 28.7, 27.1, 26.0, 19.5, 9.1. HRMS (ESI, *m*/*z*): calculated for [M + H]^+^ C_22_H_29_OSi 337.1982, found: 339.1956.

### 3.6. Synthesis of (2S,7S)-7-((tert-butyldiphenylsilyl)oxy)non-4-yn-2-ol ((2S,7S)-***7***)

Under an argon atmosphere, *n*-BuLi (2.69 mL, 2.4 M in *n*-hexane, 6.45 mmol, 2.15 equiv.) was added dropwise to a stirred solution of (*S*)-tert-butyl(hex-5-yn-3-yloxy)diphenylsilane ((*S*)-**5**) (2.54 g, 7.50 mmol, 2.5 equiv.) in THF (15 mL) at −78 °C. The mixture was stirred for 1.5 h at the same temperature, and a solution of (*S*)-2-methyloxirane ((*S*)-**6**) (>99% ee, 174.3 mg, 3.00 mmol, 1.0 equiv.) in THF (1 mL) and boron trifluoride-diethyl etherate (852.0 mg, 6.00 mmol, 2.0 equiv.), which were pre-chilled at −78 °C, was then added. The reaction mixture was allowed to warm to room temperature and stirred continuously for 12 h. The reaction was quenched by adding saturated aqueous NaHCO_3_ solution (5 mL), followed by separation of the organic phase. The aqueous phase was extracted with EtOAc (3 × 10 mL). The combined organic layers were washed with brine (10 mL), dried over anhydrous Na_2_SO_4_ and concentrated under reduced pressure to yield the crude product. The crude product was purified by silica gel column chromatography using an eluent of ethyl acetate and petroleum ether (1:5) to produce (2*S*,7*S*)-7-((tert-butyldiphenylsilyl)oxy)non-4-yn-2-ol ((2*S*,7*S*)-**7**) (1.15 g, 97% yield) as a colorless oil. [*α*]_D_^25^ = −28.17 (c 1.53, CHCl_3_). ^1^H NMR (500 MHz, CDCl_3_) δ 7.63–7.60 (m, 4H), 7.35–7.28 (m, 6H), 3.80–3.74 (m, 1H), 3.72–3.67 (m, 1H), 2.26–2.12 (m, 4H), 1.69 (br s, 1H), 1.57–1.49 (m, 2H), 1.13 (d, *J* = 6.2 Hz, 3H), 0.99 (s, 9H), 0.77 (t, *J* = 7.5 Hz, 3H). ^13^C NMR (125 MHz, CDCl_3_) δ 136.0, 134.5, 134.3, 129.73, 129.69, 127.7, 127.6, 80.2, 78.0, 73.0, 66.6, 29.6, 29.0, 27.1, 26.3, 22.3, 19.5, 9.2. HRMS (ESI, *m*/*z*): calculated for [M + H]^+^ C_25_H_35_O_2_Si 395.2401, found: 395.2396.

### 3.7. Synthesis of (2R,7S)-7-((tert-butyldiphenylsilyl)oxy)non-4-yn-2-ol ((2R,7S)-***7***)

Using a similar procedure for chiral TBDPS ether alcohol (2*S*,7*S*)-**7**, the ring opening of (*R*)-2-methyloxirane (*R*)-**6** (>99% ee, 174.3 mg, 3.00 mmol, 1.0 equiv.) with (*S*)-tert-butyl(hex-5-yn-3-yloxy)diphenylsilane (*S*)-**5** (2.54 g, 7.50 mmol, 2.5 equiv.) and *n*-BuLi (2.69 mL, 2.4 M in *n*-hexane, 6.45 mmol, 2.15 equiv.) yielded (2*R*,7*S*)-7-((tert-butyldiphenylsilyl)oxy)non-4-yn-2-ol ((2*R*,7*S*)-**7**) (1.15 g, 97% yield) as a colorless oil. [*α*]_D_^25^ = −30.66 (c 4.84, CHCl_3_). ^1^H NMR (500 MHz, CDCl_3_) δ 7.70–7.67 (m, 4H), 7.43–7.35 (m, 6H), 3.87–3.81 (m, 1H), 3.80–3.75 (m, 1H), 2.33–2.20 (m, 4H), 1.99 (br s, 1H), 1.65–1.56 (m, 2H), 1.20 (d, *J* = 6.1 Hz, 3H), 1.06 (s, 9H), 0.84 (t, *J* = 7.5 Hz, 3H). ^13^C NMR (125 MHz, CDCl_3_) δ 136.0, 134.5, 134.2, 129.72, 129.68, 127.7, 127.6, 80.2, 78.0, 73.0, 66.6, 29.6, 28.9, 27.1, 26.3, 22.3, 19.5, 9.2. HRMS (ESI, *m*/*z*): calculated for [M + H]^+^ C_25_H_35_O_2_Si 395.2401, found: 395.2401.

### 3.8. Synthesis of (2S,7R)-7-((tert-butyldiphenylsilyl)oxy)non-4-yn-2-ol ((2S,7R)-***7***)

Using a similar procedure for chiral TBDPS ether alcohol (2*S*,7*S*)-**7**, the ring opening of (*S*)-2-methyloxirane ((*S*)-**6**) (>99% ee, 174.3 mg, 3.00 mmol, 1.0 equiv.) with (*R*)-tert-butyl(hex-5-yn-3-yloxy)diphenylsilane ((*R*)-**5**) (2.54 g, 7.50 mmol, 2.5 equiv.) and *n*-BuLi (2.69 mL, 2.4 M in *n*-hexane, 6.45 mmol, 2.15 equiv.) yielded (2*S*,7*R*)-7-((tert-butyldiphenylsilyl)oxy)non-4-yn-2-ol ((2*S*,7*R*)-**7**) (1.17 g, 99% yield) as a light yellow oil. [*α*]_D_^25^ = +32.25 (c 3.28, CHCl_3_). ^1^H NMR (500 MHz, CDCl_3_) δ 7.70–7.67 (m, 4H), 7.43–7.35 (m, 6H), 3.85–3.82 (m, 1H), 3.79–3.75 (m, 1H), 2.33–2.19 (m, 4H), 1.98 (br s, 1H), 1.64–1.57 (m, 2H), 1.20 (d, *J* = 6.1 Hz, 3H), 1.06 (s, 9H), 0.84 (t, *J* = 7.5 Hz, 3H). ^13^C NMR (125 MHz, CDCl_3_) δ 136.0, 134.5, 134.2, 129.72, 129.69, 127.7, 127.6, 80.2, 78.0, 73.0, 66.6, 29.6, 28.9, 27.1, 26.3, 22.3, 19.5, 9.2. HRMS (ESI, *m*/*z*): calculated for [M + H]^+^ C_25_H_35_O_2_Si 395.2401, found: 395.2401.

### 3.9. Synthesis of (2R,7R)-7-((tert-butyldiphenylsilyl)oxy)non-4-yn-2-ol ((2R,7R)-***7***)

Using a similar procedure for chiral TBDPS ether alcohol (2*S*,7*S*)-**7**, the ring opening of (*R*)-2-methyloxirane ((*R*)-**6**) (>99% ee, 174.3 mg, 3.00 mmol, 1.0 equiv.) with (*R*)-tert-butyl(hex-5-yn-3-yloxy)diphenylsilane ((*R*)-**5**) (2.54 g, 7.50 mmol, 2.5 equiv.) and *n*-BuLi (2.69 mL, 2.4 M in *n*-hexane, 6.45 mmol, 2.15 equiv.) yielded (2*R*,7*R*)-7-((tert-butyldiphenylsilyl)oxy)non-4-yn-2-ol ((2*R*,7*R*)-**7**) (1.12 g, 95% yield) as a light yellow oil. [*α*]_D_^25^ = +22.59 (c 3.40, CHCl_3_). ^1^H NMR (500 MHz, CDCl_3_) δ 7.70–7.67 (m, 4H), 7.43–7.35 (m, 6H), 3.87–3.82 (m, 1H), 3.79–3.75 (m, 1H), 2.33–2.19 (m, 4H), 1.98 (br s, 1H), 1.65–1.56 (m, 2H), 1.20 (d, *J* = 6.2 Hz, 3H), 1.06 (s, 9H), 0.84 (t, *J* = 7.5 Hz, 3H). ^13^C NMR (125 MHz, CDCl_3_) δ 136.0, 134.5, 134.2, 129.73, 129.69, 127.7, 127.6, 80.1, 78.0, 73.0, 66.6, 29.6, 28.9, 27.1, 26.3, 22.3, 19.5, 9.2. HRMS (ESI, *m*/*z*): calculated for [M + H]^+^ C_25_H_35_O_2_Si 395.2401, found: 395.2385.

### 3.10. Synthesis of (2S,7S)-non-4-yne-2,7-diol ((2S,7S)-***8***)

Under an argon atmosphere, TBAF (4 mL, 1 M in THF, 4.00 mmol, 2.0 equiv.) was added slowly to a stirred solution of (2*S*,7*S*)-7-((tert-butyldiphenylsilyl)oxy)non-4-yn-2-ol ((2*S*,7*S*)-**7**) (790.0 mg, 2.00 mmol, 1.0 equiv.) in THF (5 mL) at 0 °C. The reaction mixture was allowed to warm to room temperature and stirred continuously overnight. The reaction was then quenched by adding water (5 mL), followed by separation of the organic phase. The aqueous phase was extracted with EtOAc (3 × 8 mL). The combined organic layers were washed with brine (10 mL), dried over anhydrous Na_2_SO_4_ and concentrated under reduced pressure to yield the crude product. The crude product was purified by silica gel column chromatography using an eluent of ethyl acetate and petroleum ether (1:2) to produce (2*S*,7*S*)-non-4-yne-2,7-diol ((2*S*,7*S*)-**8**) (290.0 mg, 93% yield) as a colorless oil. [*α*]_D_^25^ = +14.47 (c 2.24, CHCl_3_). ^1^H NMR (500 MHz, CDCl_3_) δ 3.89–3.84 (m, 1H), 3.61–3.56 (m, 1H), 2.44 (br s, 2H), 2.38–2.30 (m, 2H), 2.26–2.21 (m, 2H), 1.53–1.45 (m, 2H), 1.18 (d, *J* = 6.3 Hz, 3H), 0.89 (t, *J* = 7.5 Hz, 3H). ^13^C NMR (125 MHz, CDCl_3_) δ 79.3, 79.2, 71.7, 66.6, 29.4, 29.3, 27.2, 22.4, 10.1. HRMS (ESI, *m*/*z*): calculated for [M + H]^+^ C_9_H_17_O_2_ 157.1223, found: 157.1225.

### 3.11. Synthesis of (2R,7S)-non-4-yne-2,7-diol ((2R,7S)-***8***)

Using a similar procedure for chiral alkynyl diol (2*S*,7*S*)-**8**, deprotection of (2*R*,7*S*)-7-((tert-butyldiphenylsilyl)oxy)non-4-yn-2-ol ((2*R*,7*S*)-**7**) (790.0 mg, 2.00 mmol, 1.0 equiv.) with TBAF (4 mL, 1 M in THF, 4.00 mmol, 2.0 equiv.) yielded (2*R*,7*S*)-non-4-yne-2,7-diol ((2*R*,7*S*)-**8**) (284 mg, 91% yield) as a colorless oil. [*α*]_D_^25^ = −3.83 (c 1.25, CHCl_3_). ^1^H NMR (500 MHz, CDCl_3_) δ 3.96–3.90 (m, 1H), 3.68–3.63 (m, 1H), 2.46–2.28 (m, 6H), 1.59–1.53 (m, 2H), 1.25 (d, *J* = 6.2 Hz, 3H), 0.96 (t, *J* = 7.5 Hz, 3H). ^13^C NMR (125 MHz, CDCl_3_) δ 79.3, 79.2, 71.7, 66.6, 29.4, 29.3, 27.3, 22.4, 10.1. HRMS (ESI, *m*/*z*): calculated for [M + H]^+^ C_9_H_17_O_2_ 157.1223, found: 157.1220.

### 3.12. Synthesis of (2S,7R)-non-4-yne-2,7-diol ((2S,7R)-***8***)

Using a similar procedure for chiral alkynyl diol (2*S*,7*S*)-**8**, deprotection of (2*S*,7*R*)-7-((tert-butyldiphenylsilyl)oxy)non-4-yn-2-ol ((2*S*,7*R*)-**7**) (790.0 mg, 2.00 mmol, 1.0 equiv.) with TBAF (4 mL, 1 M in THF, 4.00 mmol, 2.0 equiv.) yielded (2*S*,7*R*)-non-4-yne-2,7-diol ((2*S*,7*R*)-**8**) (264 mg, 86% yield) as a colorless oil. [*α*]_D_^25^ = +3.18 (c 2.14, CHCl_3_). ^1^H NMR (500 MHz, CDCl_3_) δ 3.96–3.90 (m, 1H), 3.67–3.64 (m, 1H), 2.62–2.28 (m, 6H), 1.59–1.54 (m, 2H), 1.25 (d, *J* = 6.2 Hz, 3H), 0.96 (t, *J* = 7.5 Hz, 3H). ^13^C NMR (125 MHz, CDCl_3_) δ 79.3, 79.2, 71.7, 66.6, 29.4, 29.2, 27.2, 22.4, 10.0. HRMS (ESI, *m*/*z*): calculated for [M + H]^+^ C_9_H_17_O_2_ 157.1223, found: 157.1221.

### 3.13. Synthesis of (2R,7R)-non-4-yne-2,7-diol ((2R,7R)-***8***)

Using a similar procedure for chiral alkynyl diol (2*S*,7*S*)-**8**, deprotection of (2*R*,7*R*)-7-((tert-butyldiphenylsilyl)oxy)non-4-yn-2-ol ((2*R*,7*R*)-**7**) (790.0 mg, 2.00 mmol, 1.0 equiv.) with TBAF (4 mL, 1 M in THF, 4.00 mmol, 2.0 equiv.) yielded (2*R*,7*R*)-non-4-yne-2,7-diol ((2*R*,7*R*)-**8**) (293 mg, 94% yield) as a colorless oil. [*α*]_D_^25^ = −20.82 (c 1.13, CHCl_3_). ^1^H NMR (500 MHz, CDCl_3_) δ 3.97–3.90 (m, 1H), 3.68–3.63 (m, 1H), 2.46–2.27 (m, 6H), 1.59–1.53 (m, 2H), 1.25 (d, *J* = 6.3 Hz, 3H), 0.96 (t, *J* = 7.5 Hz, 3H). ^13^C NMR (125 MHz, CDCl_3_) δ 79.4, 79.2, 71.7, 66.6, 29.4, 29.3, 27.3, 22.5, 10.1. HRMS (ESI, *m*/*z*): calculated for [M + H]^+^ C_9_H_17_O_2_ 157.1223, found: 157.1220.

### 3.14. Synthesis of (2S,7S)-nonane-2,7-diol ((2S,7S)-***9***)

Under a hydrogen atmosphere, (2*S*,7*S*)-non-4-yne-2,7-diol ((2*S*,7*S*)-**8**) (180.0 mg, 1.15 mmol, 1.0 equiv.) was add to a stirred solution of Pd/C (18.0 mg, 10%) in MeOH (5 mL) at room temperature. The reaction mixture was stirred continuously overnight under a hydrogen atmosphere, followed by being filtered through diatomaceous earth. The filtrate was concentrated under reduced pressure to yield the crude product. The crude product was purified by silica gel column chromatography using an eluent of ethyl acetate and petroleum ether (1:2) to produce (2*S*,7*S*)-nonane-2,7-diol ((2*S*,7*S*)-**9**) (162.0 mg, 88% yield) as a colorless oil. [*α*]_D_^25^ = +14.77 (c 0.87, CHCl_3_). ^1^H NMR (500 MHz, CDCl_3_) δ 3.77–3.71 (m, 1H), 3.49–3.44 (m, 1H), 1.58 (br s, 2H), 1.47–1.30 (m, 10H), 1.12 (d, *J* = 6.2 Hz, 3H), 0.87 (t, *J* = 7.5 Hz, 3H). ^13^C NMR (125 MHz, CDCl_3_) δ 73.3, 68.1, 39.4, 37.0, 30.3, 25.9, 25.7, 23.7, 10.0. HRMS (ESI, *m*/*z*): calculated for [M + H]^+^ C_9_H_21_O_2_ 161.1536, found: 161.1538.

### 3.15. Synthesis of (2R,7S)-nonane-2,7-diol ((2R,7S)-***9***)

Using a similar procedure for chiral diol (2*S*,7*S*)-**9**, the hydrogenation of (2*R*,7*S*)-non-4-yne-2,7-diol ((2*R*,7*S*)-**8**) (154.0 mg, 0.99 mmol, 1.0 equiv.) catalyzed with Pd/C (15.0 mg, 10%) yielded (2*R*,7*S*)-nonane-2,7-diol ((2*R*,7*S*)-**9**) (145.0 mg, 92% yield) as a colorless oil. [*α*]_D_^25^ = +0.56 (c 2.15, CHCl_3_). ^1^H NMR (500 MHz, CDCl_3_) δ 3.83–3.77 (m, 1H), 3.55–3.51 (m, 1H), 1.71 (br s, 2H), 1.53–1.40 (m, 8H), 1.37–1.30 (m, 2H), 1.19 (d, *J* = 6.2 Hz, 3H), 0.94 (t, *J* = 7.5 Hz, 3H). ^13^C NMR (125 MHz, CDCl_3_) δ 73.3, 68.1, 39.4, 36.9, 30.3, 25.9, 25.8, 23.6, 10.0. HRMS (ESI, *m*/*z*): calculated for [M + H]^+^ C_9_H_21_O_2_ 161.1536, found: 161.1538.

### 3.16. Synthesis of (2S,7R)-nonane-2,7-diol ((2S,7R)-***9***)

Using a similar procedure for chiral diol (2*S*,7*S*)-**9**, the hydrogenation of (2*S*,7*R*)-non-4-yne-2,7-diol ((2*S*,7*R*)-**8**) (180.0 mg, 1.15 mmol, 1.0 equiv.) catalyzed with Pd/C (18.0 mg, 10%) yielded (2*S*,7*R*)-nonane-2,7-diol ((2*S*,7*R*)-**9**) (170.0 mg, 92% yield) as a colorless oil. [*α*]_D_^25^ = −1.24 (c 1.93, CHCl_3_). ^1^H NMR (500 MHz, CDCl_3_) δ 3.75–3.69 (m, 1H), 3.46–3.43 (m, 1H), 1.85 (br s, 2H), 1.46–1.33 (m, 8H), 1.31–1.24 (m, 2H), 1.11 (d, *J* = 6.2 Hz, 3H), 0.87 (t, *J* = 7.5 Hz, 3H). ^13^C NMR (125 MHz, CDCl_3_) δ 73.3, 68.1, 39.3, 36.9, 30.3, 25.9, 25.8, 23.6, 10.0. HRMS (ESI, *m*/*z*): calculated for [M + H]^+^ C_9_H_21_O_2_ 161.1536, found: 161.1539.

### 3.17. Synthesis of (2R,7R)-nonane-2,7-diol ((2R,7R)-***9***)

Using a similar procedure for chiral diol (2*S*,7*S*)-**9**, the hydrogenation of (2*R*,7*R*)-non-4-yne-2,7-diol (2*R*,7*R*)-**8** (293.0 mg, 1.87 mmol, 1.0 equiv.) catalyzed with Pd/C (30.0 mg, 10%) yielded (2*R*,7*R*)-nonane-2,7-diol ((2*R*,7*R*)-**9**) (270.0 mg, 90% yield) as a colorless oil. [*α*]_D_^25^ = −17.76 (c 1.25, CHCl_3_). ^1^H NMR (500 MHz, CDCl_3_) δ 3.76–3.71 (m, 1H), 3.48–3.43 (m, 1H), 1.65 (br s, 2H), 1.47–1.27 (m, 10H), 1.12 (d, *J* = 6.2 Hz, 3H), 0.87 (t, *J* = 7.5 Hz, 3H). ^13^C NMR (125 MHz, CDCl_3_) δ 73.3, 68.1, 39.3, 36.9, 30.3, 25.8, 25.7, 23.6, 10.0. HRMS (ESI, *m*/*z*): calculated for [M + H]^+^ C_9_H_21_O_2_ 161.1536, found: 161.1538.

### 3.18. Synthesis of (2S,7S)-nonane-2,7-diyl dibutyrate ((2S,7S)-***1***)

Under an argon atmosphere, butyric anhydride (**10**) (95.0 mg, 0.60 mmol, 3.0 equiv.) was added slowly to a stirred solution of (2*S*,7*S*)-nonane-2,7-diol ((2*S*,7*S*)-**9**) (32.0 mg, 0.20 mmol, 1.0 equiv.) in anhydrous pyridine (5 mL) at 0 °C. The reaction solution was allowed to warm to room temperature and stirred continuously overnight. The mixture was concentrated under reduced pressure to yield the crude product. The crude product was purified by silica gel column chromatography using an eluent of ethyl acetate and petroleum ether (1:50) to produce (2*S*,7*S*)-nonane-2,7-diyl dibutyrate ((2*S*,7*S*)-**1**) (56.0 mg, 94% yield) as a colorless oil. [*α*]_D_^25^ = −2.41 (c 1.16, CHCl_3_). Lit. [20] [*α*]_D_^20^ =–6.7 (c 0.6, CHCl_3_). ^1^H NMR (500 MHz, CDCl_3_) δ 4.92–4.86 (m, 1H), 4.84–4.79 (m, 1H), 2.26 (dt, *J* = 11.3, 7.5 Hz, 4H), 1.69–1.61 (m, 4H), 1.57–1.44 (m, 6H), 1.33–1.26 (m, 4H), 1.19 (d, *J* = 6.3 Hz, 3H), 0.97–0.87 (m, 6H), 0.87 (t, *J* = 7.5 Hz, 3H). ^13^C NMR (125 MHz, CDCl_3_) δ 173.7, 173.5, 75.2, 70.7, 36.8, 36.7, 36.0, 33.7, 27.1, 25.5, 25.3, 20.1, 18.8, 18.7, 13.83, 13.79, 9.7. HRMS (ESI, *m*/*z*): calculated for [M + H]^+^ C_17_H_33_O_4_ 301.2373, found: 301.2374.

### 3.19. Synthesis of (2R,7S)-nonane-2,7-diyl dibutyrate ((2R,7S)-***1***)

Using a similar procedure for pheromone (2*S*,7*S*)-**1**, the acylation of (2*R*,7*S*)-nonane-2,7-diol ((2*R*,7*S*)-**9**) (135.0 mg, 0.84 mmol, 1.0 equiv.) with butyric anhydride (**10**) (400.0 mg, 2.53 mmol, 3.0 equiv.) yielded (2*R*,7*S*)-nonane-2,7-diyl dibutyrate ((2*R*,7*S*)-**1**) (237.0 mg, 94% yield) as a colorless oil. [*α*]_D_^25^ = −3.97 (c 1.00, CHCl_3_). ^1^H NMR (500 MHz, CDCl_3_) δ 4.92–4.86 (m, 1H), 4.84–4.79 (m, 1H), 2.26 (dt, *J* = 11.3, 7.4 Hz, 4H), 1.69–1.61 (m, 4H), 1.58–1.46 (m, 6H), 1.35–1.26 (m, 4H), 1.19 (d, *J* = 6.2 Hz, 3H), 0.97–0.93 (m, 6H), 0.87 (t, *J* = 7.5 Hz, 3H). ^13^C NMR (125 MHz, CDCl_3_) δ 173.7, 173.5, 75.2, 70.7, 36.8, 36.7, 36.0, 33.7, 27.1, 25.5, 25.3, 20.1, 18.8, 18.7, 13.83, 13.78, 9.7. HRMS (ESI, *m*/*z*): calculated for [M + H]^+^ C_17_H_33_O_4_ 301.2373, found: 301.2375.

### 3.20. Synthesis of (2S,7R)-nonane-2,7-diyl dibutyrate ((2S,7R)-***1***)

Using a similar procedure for pheromone (2*S*,7*S*)-**1**, the acylation of (2*S*,7*R*)-nonane-2,7-diol ((2*S*,7*R*)-**9**) (127.0 mg, 0.79 mmol, 1.0 equiv.) with butyric anhydride (**10**) (376.0 mg, 2.38 mmol, 3.0 equiv.) yielded (2*S*,7*R*)-nonane-2,7-diyl dibutyrate ((2*S*,7*R*)-**1**) (221.0 mg, 93% yield) as a colorless oil. [*α*]_D_^25^ = +3.46 (c 1.97, CHCl_3_). ^1^H NMR (500 MHz, CDCl_3_) δ 4.92–4.86 (m, 1H), 4.84–4.79 (m, 1H), 2.26 (dt, *J* = 11.3, 7.4 Hz, 4H), 1.69–1.61 (m, 4H), 1.59–1.46 (m, 6H), 1.36–1.24 (m, 4H), 1.19 (d, *J* = 6.3 Hz, 3H), 0.97–0.93 (m, 6H), 0.87 (t, *J* = 7.5 Hz, 3H). ^13^C NMR (125 MHz, CDCl_3_) δ 173.7, 173.5, 75.1, 70.7, 36.74, 36.72, 36.0, 33.7, 27.1, 25.5, 25.3, 20.1, 18.8, 18.7, 13.82, 13.77, 9.7. HRMS (ESI, *m*/*z*): calculated for [M + H]^+^ C_17_H_33_O_4_ 301.2373, found: 301.2374.

### 3.21. Synthesis of (2R,7R)-nonane-2,7-diyl dibutyrate ((2R,7R)-***1***)

Using a similar procedure for pheromone (2*S*,7*S*)-**1**, the acylation of (2*R*,7*R*)-nonane-2,7-diol ((2*R*,7*R*)-**9**) (258.0 mg, 1.61 mmol, 1.0 equiv.) with butyric anhydride (**10**) (764.0 mg, 4.83 mmol, 3.0 equiv.) yielded (2*R*,7*R*)-nonane-2,7-diyl dibutyrate ((2*R*,7*R*)-**1**) (458.0 mg, 95% yield) as a colorless oil. [*α*]_D_^25^ = +2.37 (c 1.52, CHCl_3_). ^1^H NMR (500 MHz, CDCl_3_) δ 4.92–4.86 (m, 1H), 4.84–4.79 (m, 1H), 2.26 (dt, *J* = 11.3, 7.4 Hz, 4H), 1.69–1.61 (m, 4H), 1.59–1.44 (m, 6H), 1.32–1.28 (m, 4H), 1.19 (d, *J* = 6.3 Hz, 3H), 0.97–0.93 (m, 6H), 0.87 (t, *J* = 7.4 Hz, 3H). ^13^C NMR (125 MHz, CDCl_3_) δ 173.7, 173.5, 75.2, 70.7, 36.74, 36.72, 36.0, 33.7, 27.1, 25.5, 25.3, 20.1, 18.8, 18.7, 13.83, 13.78, 9.7. HRMS (ESI, *m*/*z*): calculated for [M + H]^+^ C_17_H_33_O_4_ 301.2373, found: 301.2366.

## 4. Conclusions

In summary, we have conducted a new and efficient synthesis of the sex pheromone of *S. mosellana* and its stereoisomers with overall yields of 59–64%. The key reactions were the ring opening of chiral epoxide with an alkynyllithium and the hydrogenation of a triple bond. Compared with the existing synthetic methods, our approach has the advantages of cheap materials, high total yields and being easily scaled. Furthermore, our synthesis would be useful in integrated pest management programs for the orange wheat blossom midge.

## Data Availability

The data presented in this article are available in the Supplementary Materials.

## Supplementary Materials

The following supporting information can be downloaded at: https://www.mdpi.com/article/10.3390/molecules30030671/s1, Figures S1–S40 and Table S1. The ^1^H NMR and ^13^C NMR spectra for all the synthetic compounds, and comparison of NMR Data between the precious [20] and current synthesized (2*S*,7*S*)-**1.**
