# Supplementary material for: Enantioselective Synthesis of the Sex Pheromone of Sitodiplosis mosellana (Géhin) and Its Stereoisomers
Source: Molecules. 2025 Feb 3;30(3):671. doi: 10.3390/molecules30030671 (PMC11820043; doi:10.3390/molecules30030671)
Supplement: Supplementary file 1 [file molecules-30-00671-s001.zip › molecules-3425635-supplementary.pdf]

SUPPORTING INFORMATION

Enantioselective Synthesis of the Sex Pheromone *Sitodiplosis mosellana* (Géhin) And Its Stereoisomers

Jianan Wang [1], Xiaoyang Li[1], Yun Zhou[2], Qinghua Bian[1], Jiangchun Zhong [1]*

[1] Department of Applied Chemistry, China Agricultural University, Beijing 100193, P. R. China.

[2] Institute of Industrial Crops, Shandong Academy of Agricultural Sciences, Jinan 250100, P. R. China

* Correspondence: zhong@cau.edu.cn; Tel.: +86-010-6273-1356

Table of Contents

1. ^{1}H, ^{13}C NMR Spectra of the Products……………………………………………………..........................S2

2. Comparison of NMR Data Between the Precious and Current Synthesized (2*S*,7*S*)-**1**………………S22

3. References……….............……………............…………………………………………….........................S22

1. ^{1}H and ^{13}C spectra of the products

Figure S1. ^{1}H NMR Spectrum of compound (*S*)-**4** (500 MHz, $CDCl_3$).

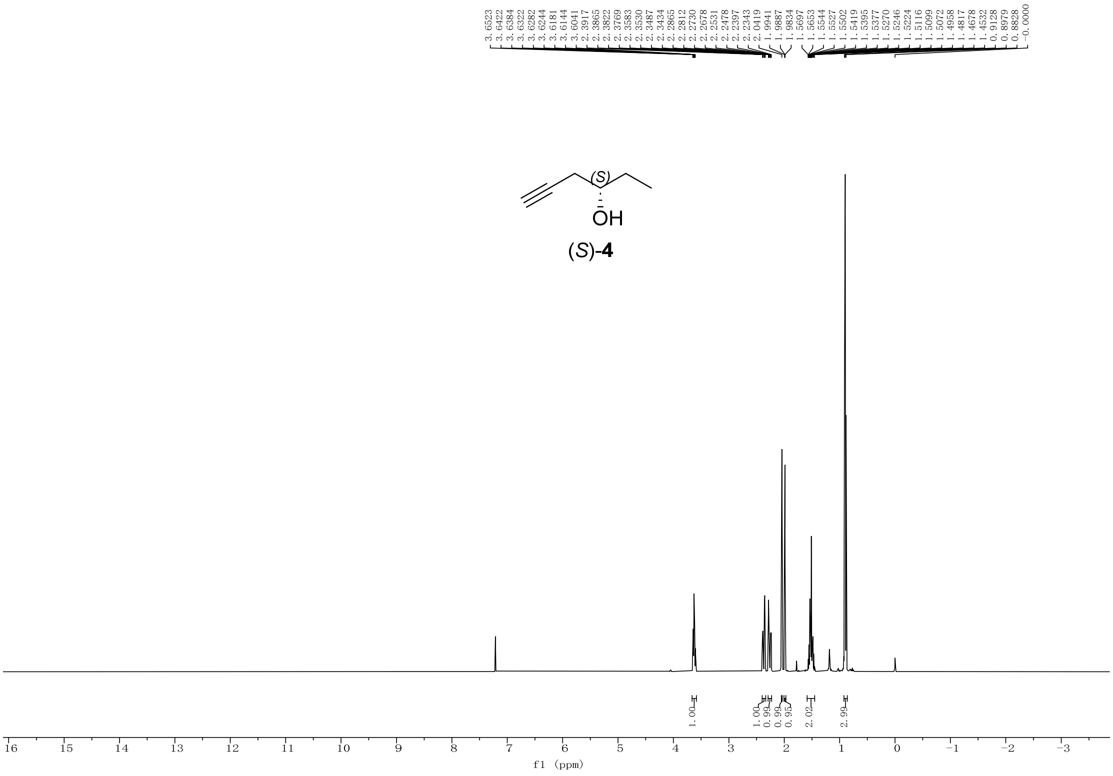

Figure S2. ^{13}C NMR Spectrum of compound (*S*)-**4** (125 MHz, $CDCl_3$).

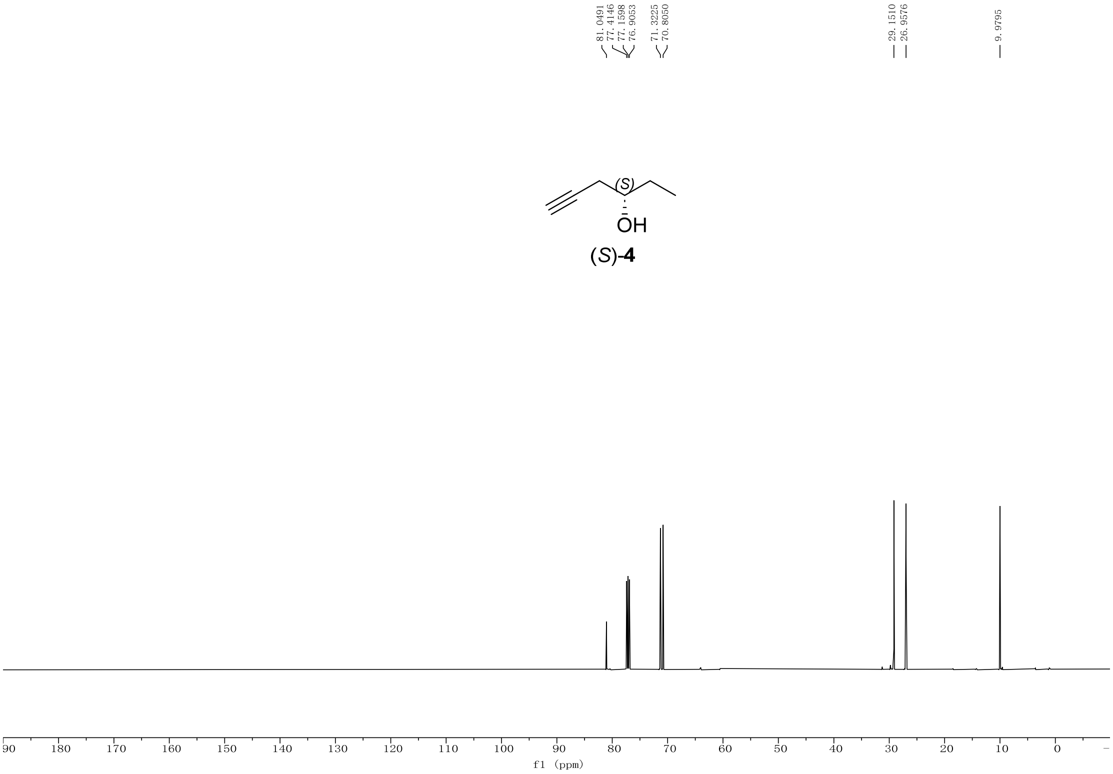

Figure S3. ^{1}H NMR Spectrum of compound (*R*)-**4** (500 MHz, $CDCl_3$).

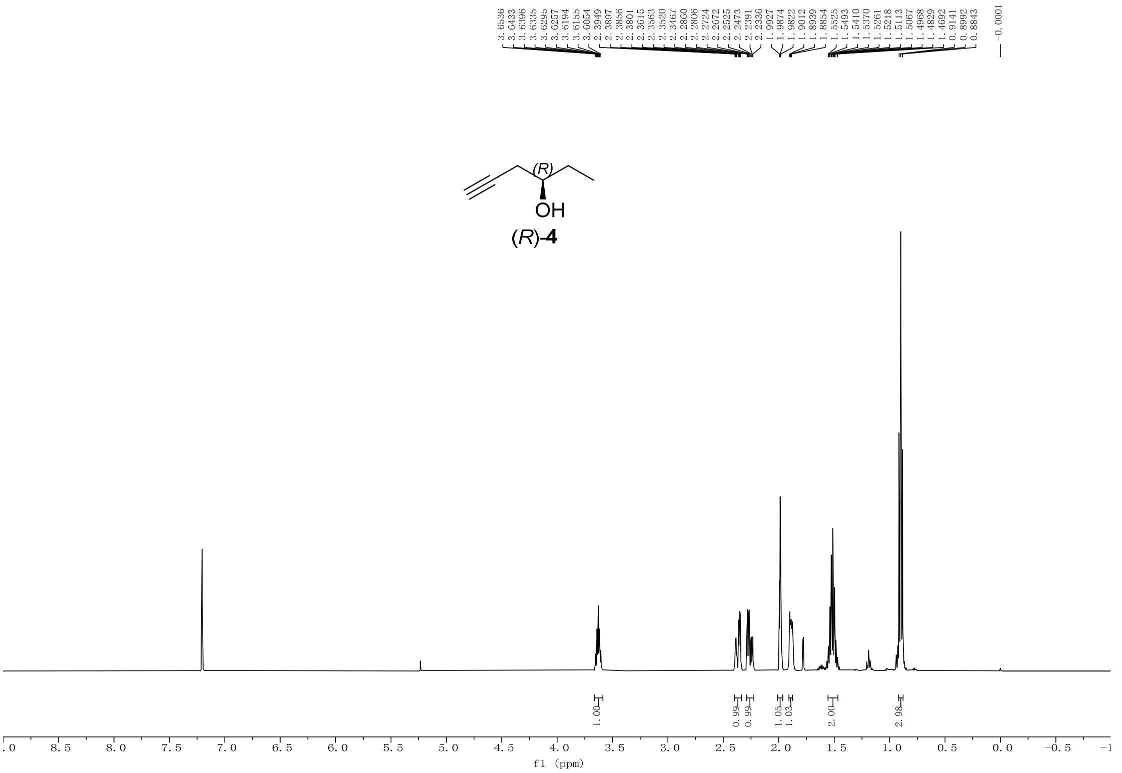

Figure S4. ^{13}C NMR Spectrum of compound (*R*)-**4** (125 MHz, $CDCl_3$).

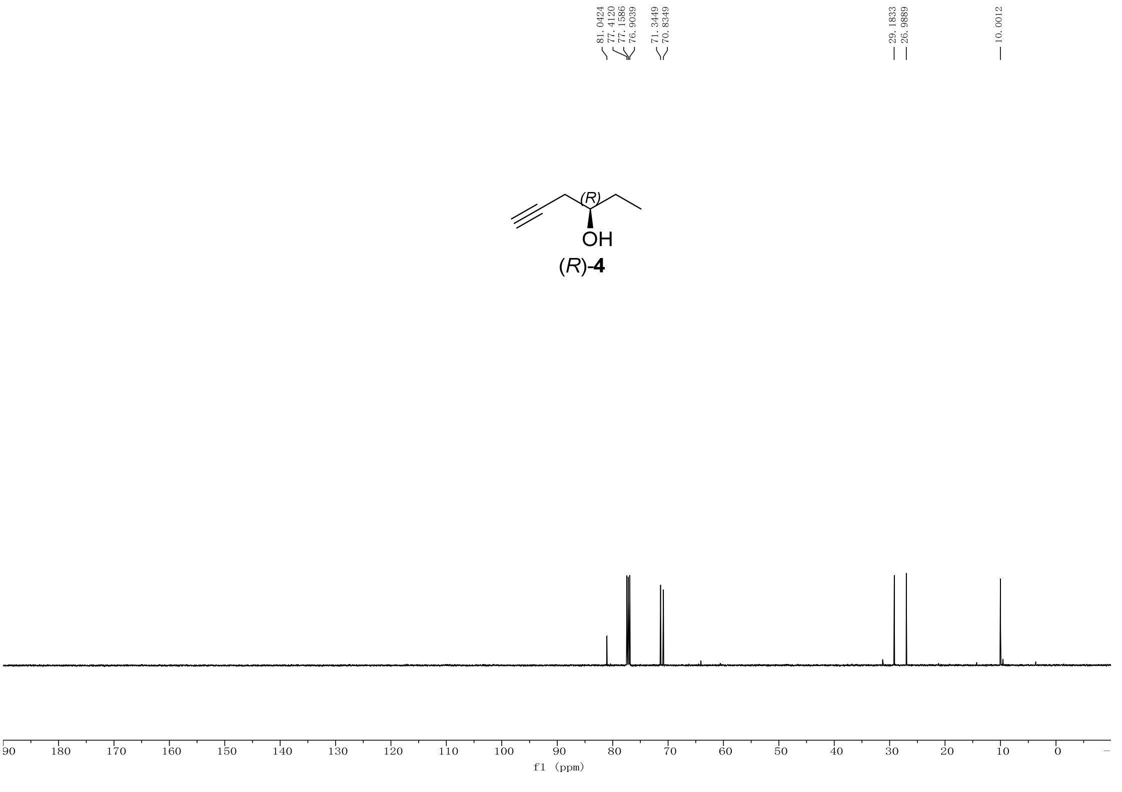

Figure S5. ^{1}H NMR Spectrum of compound (*S*)-**5** (500 MHz, $CDCl_3$).

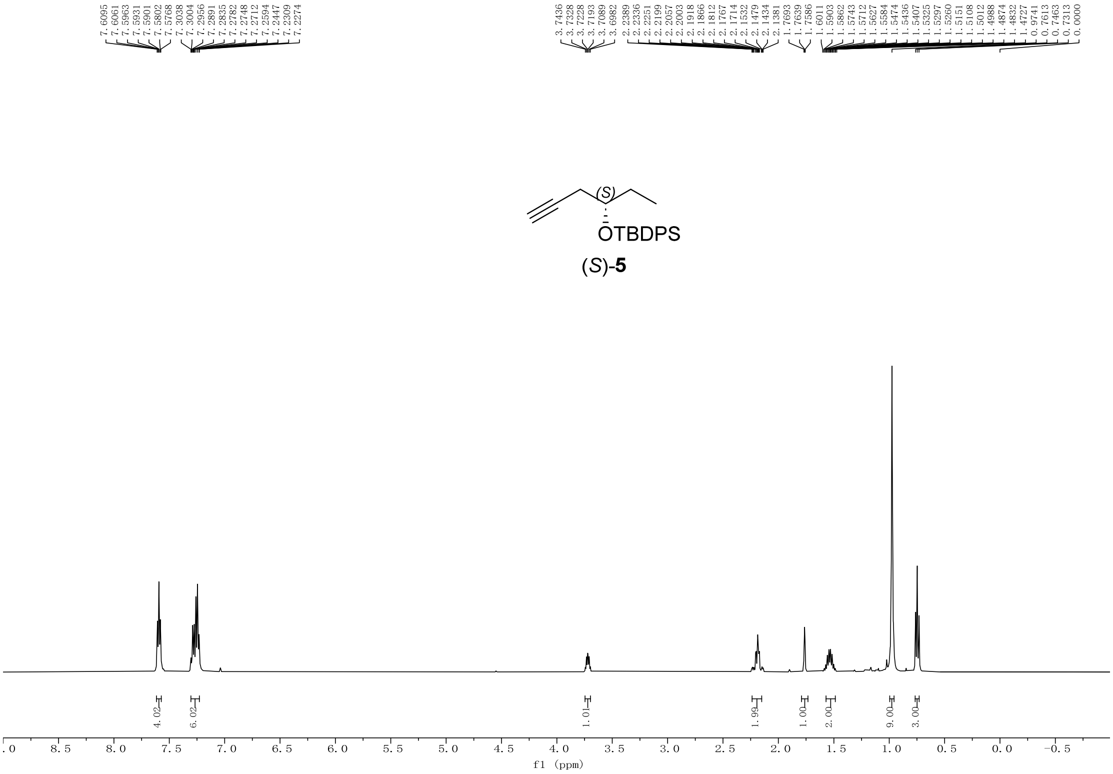

Figure S6. ^{13}C NMR Spectrum of compound (*S*)-**5** (125 MHz, $CDCl_3$).

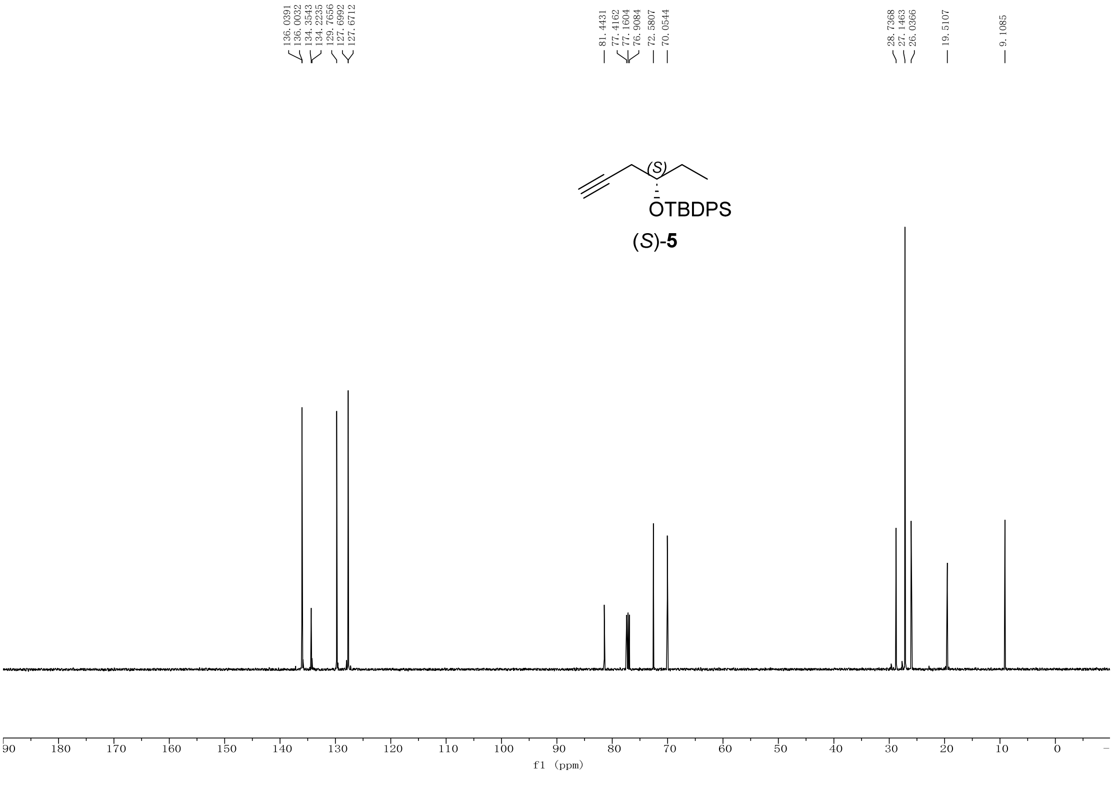

Figure S7. ^{1}H NMR Spectrum of compound (*R*)-**5** (500 MHz, CDCl$_3$).

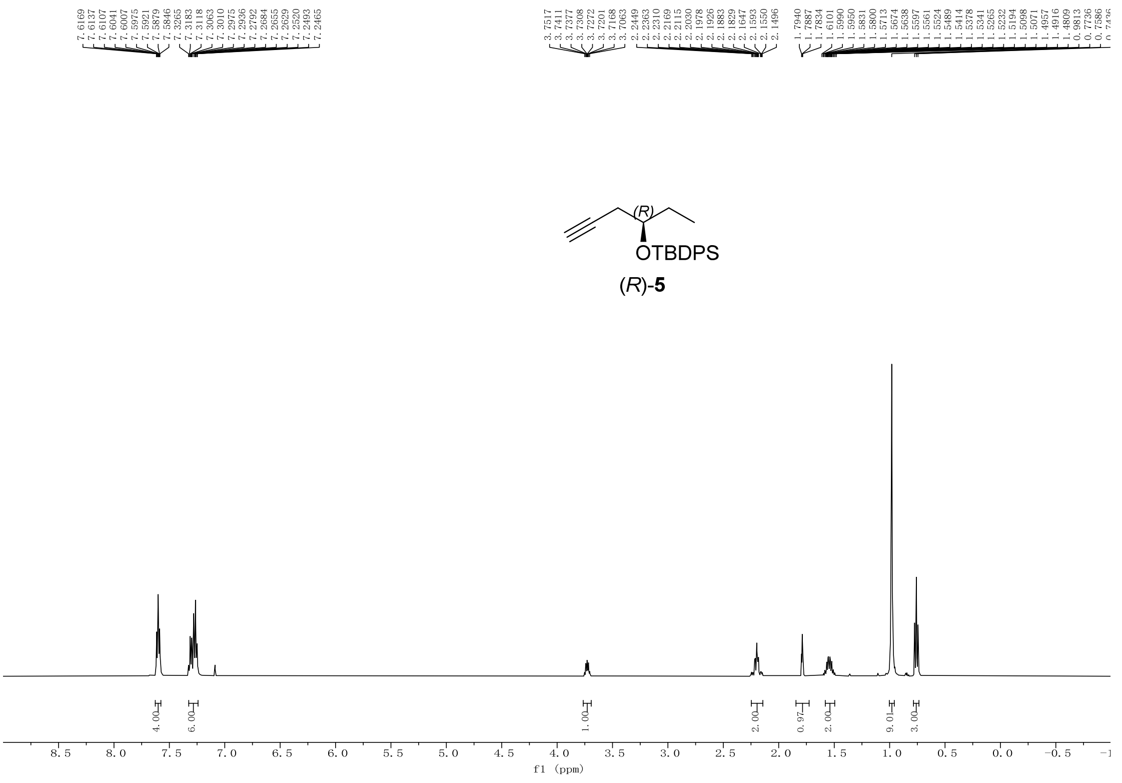

Figure S8. ^{13}C NMR Spectrum of compound (*R*)-**5** (125 MHz, CDCl$_3$).

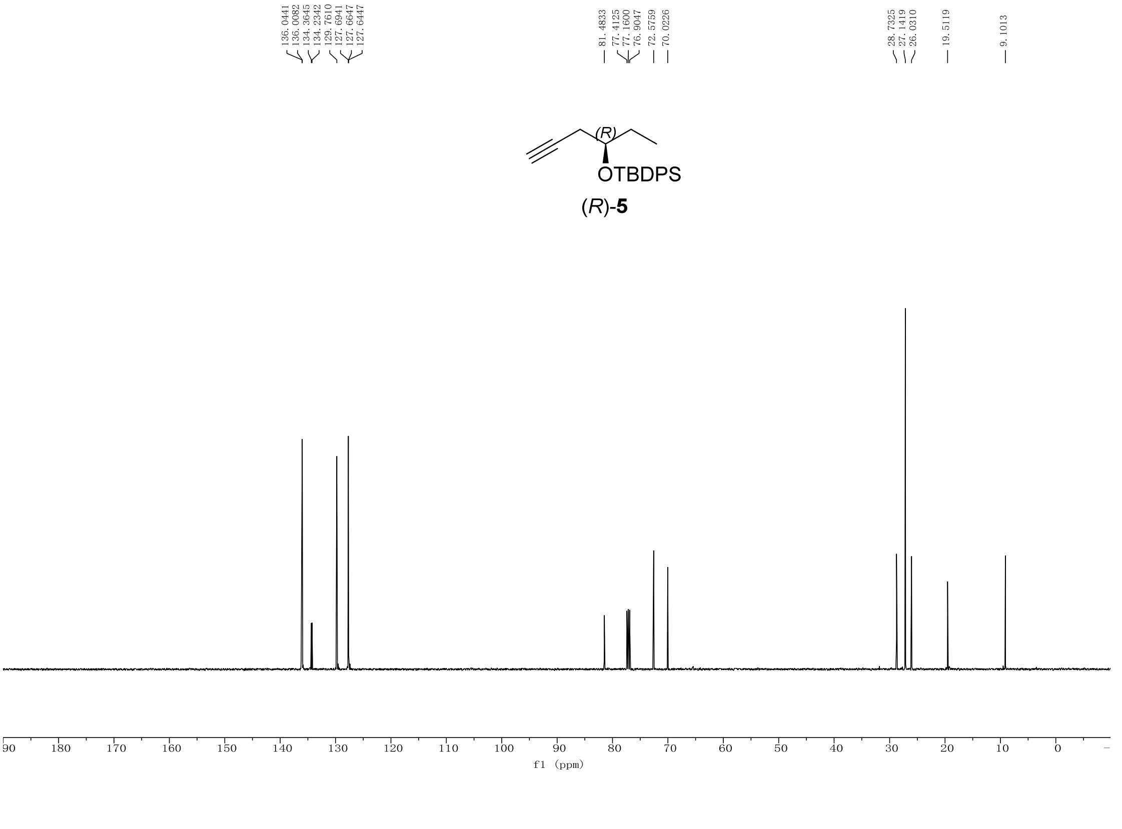

Figure S9. ^{1}H NMR Spectrum of compound (2*S*,7*S*)-**7** (500 MHz, CDCl$_3$).

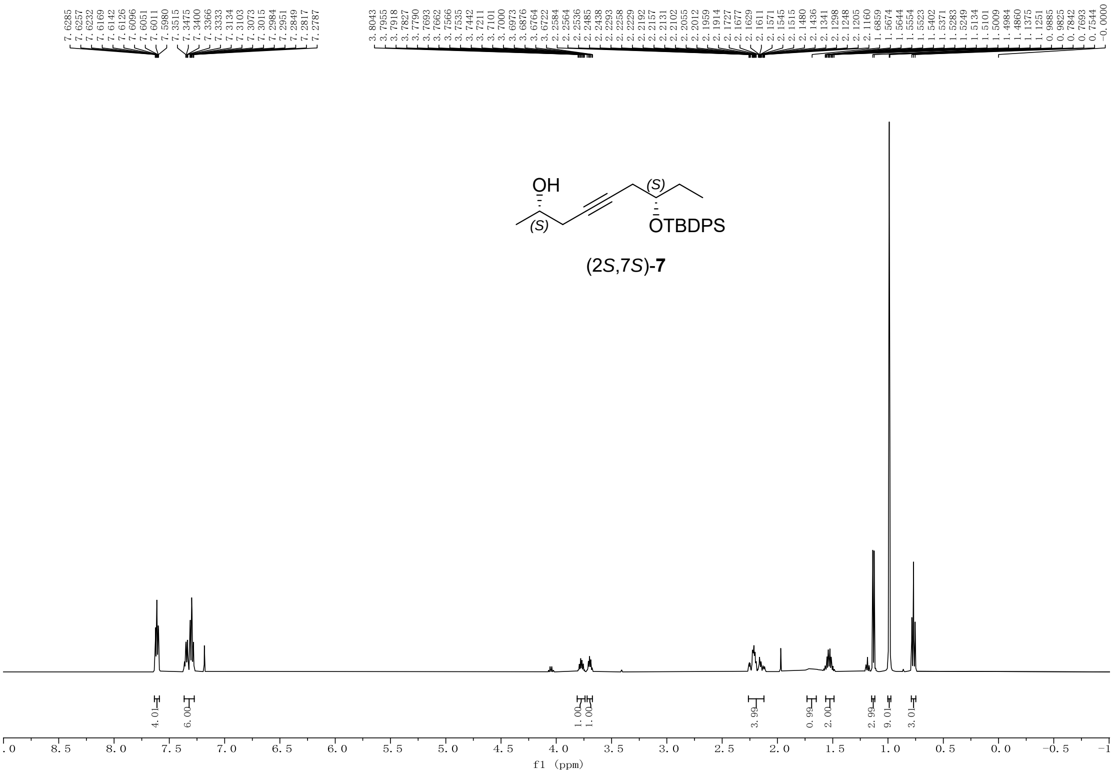

Figure S10. ^{13}C NMR Spectrum of compound (2*S*,7*S*)-**7** (125 MHz, CDCl$_3$).

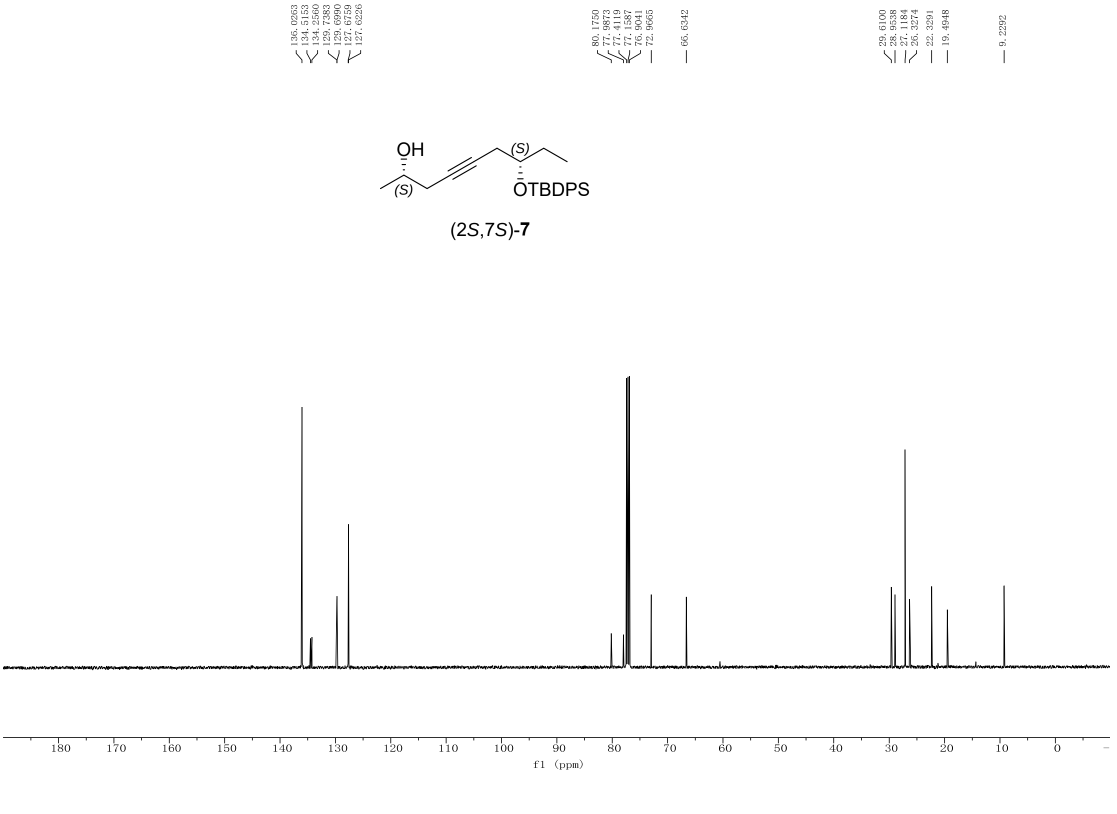

Figure S11. ^{1}H NMR Spectrum of compound (2*R*,7*S*)-**7** (500 MHz, $CDCl_3$).

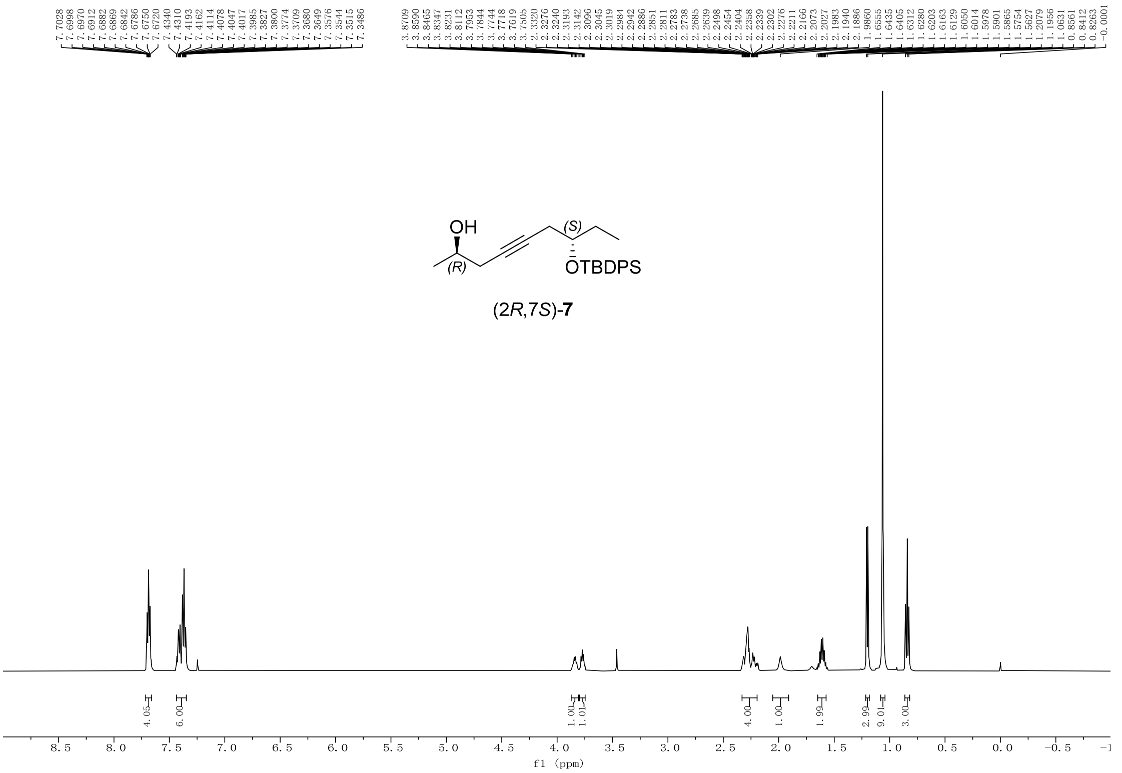

Figure S12. ^{13}C NMR Spectrum of compound (2*R*,7*S*)-**7** (125 MHz, $CDCl_3$).

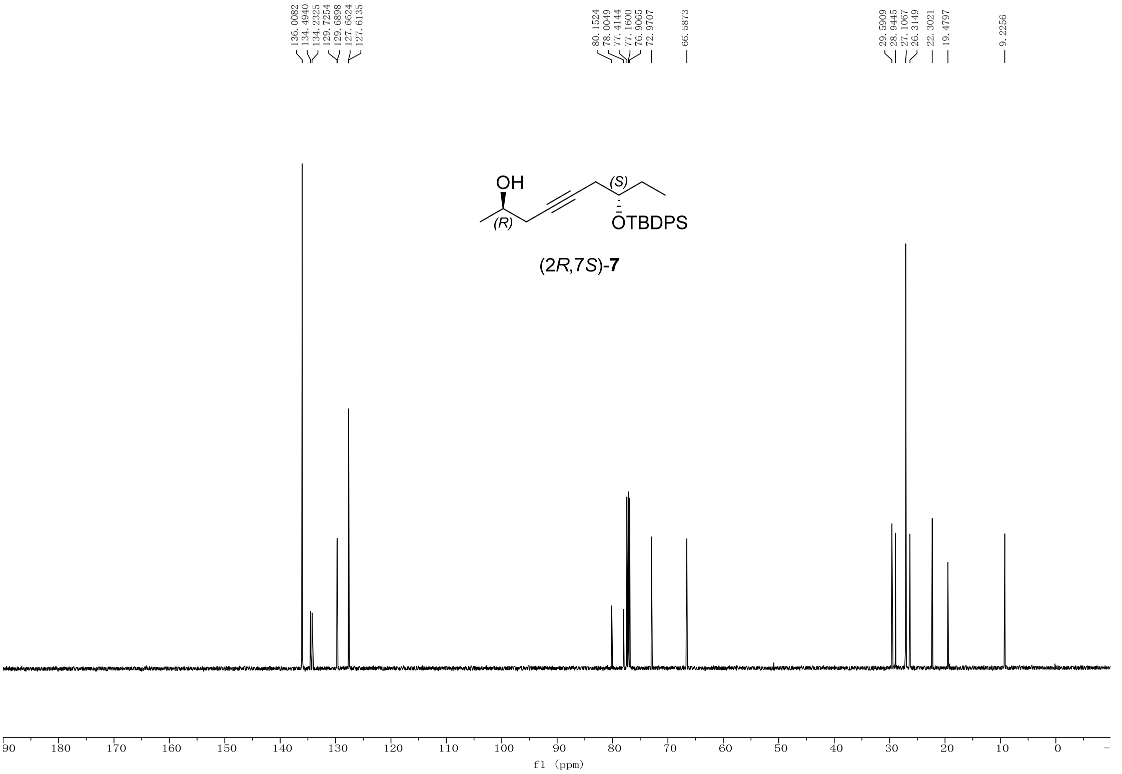

Figure S13. ^{1}H NMR Spectrum of compound (2*S*,7*R*)-**7** (500 MHz, $CDCl_3$).

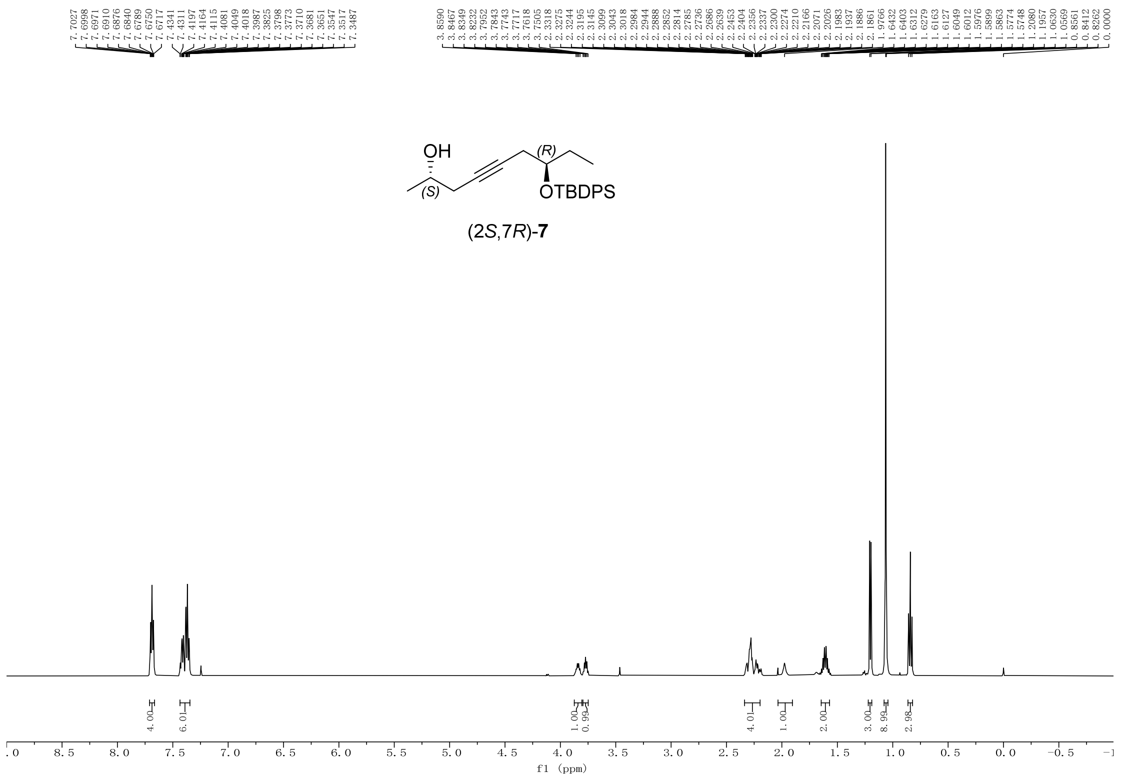

Figure S14. ^{13}C NMR Spectrum of compound (2*S*,7*R*)-**7** (125 MHz, $CDCl_3$).

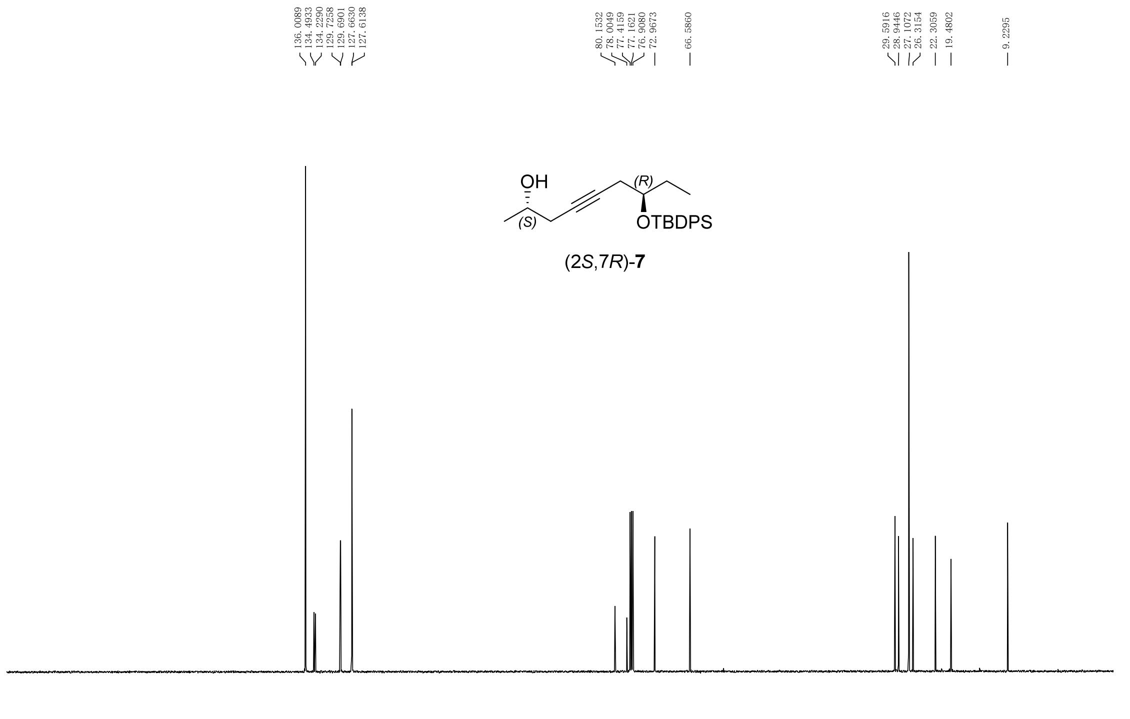

Figure S15. ^{1}H NMR Spectrum of compound (2*R*,7*R*)-**7** (500 MHz, $CDCl_3$).

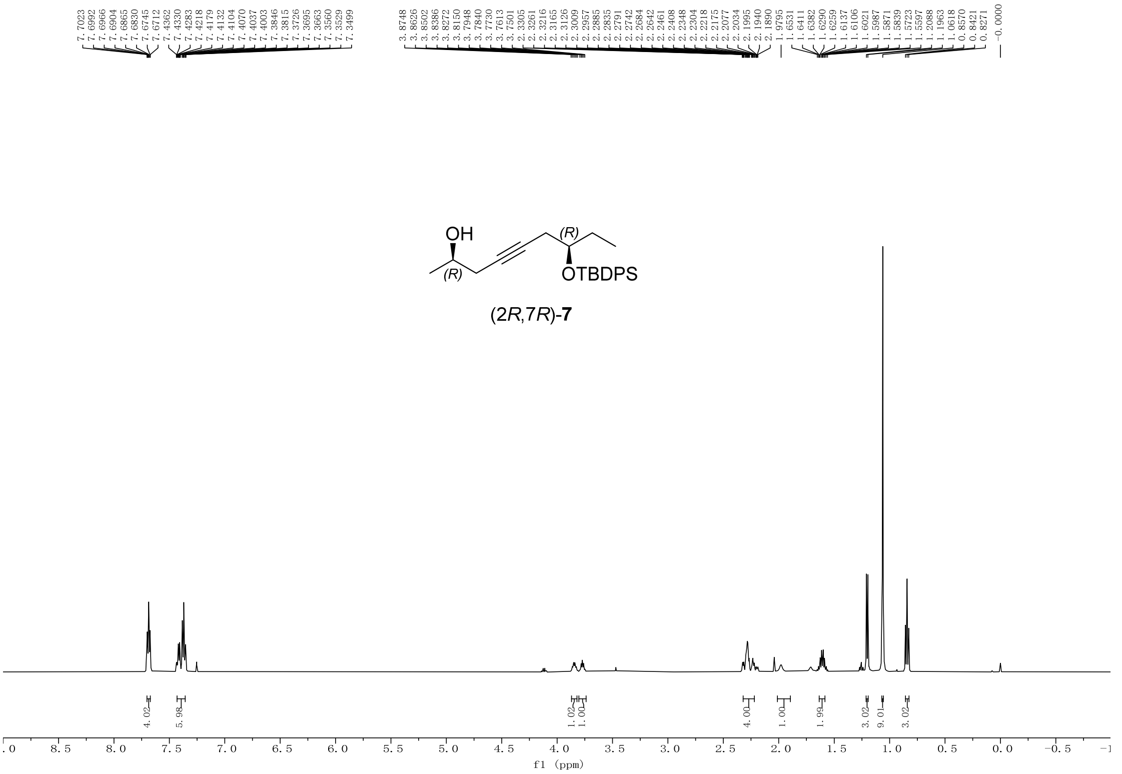

Figure S16. ^{13}C NMR Spectrum of compound (2*R*,7*R*)-**7** (125 MHz, $CDCl_3$).

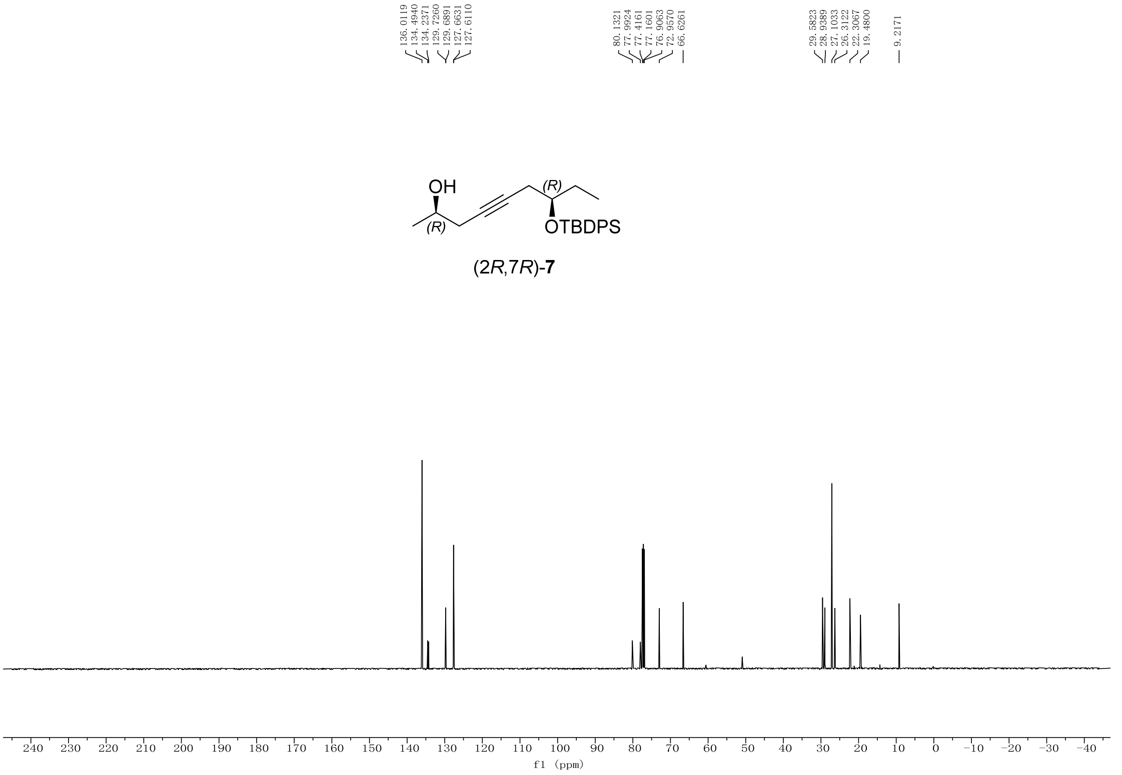

Figure S17. ^{1}H NMR Spectrum of compound (2*S*,7*S*)-**8** (500 MHz, $CDCl_3$).

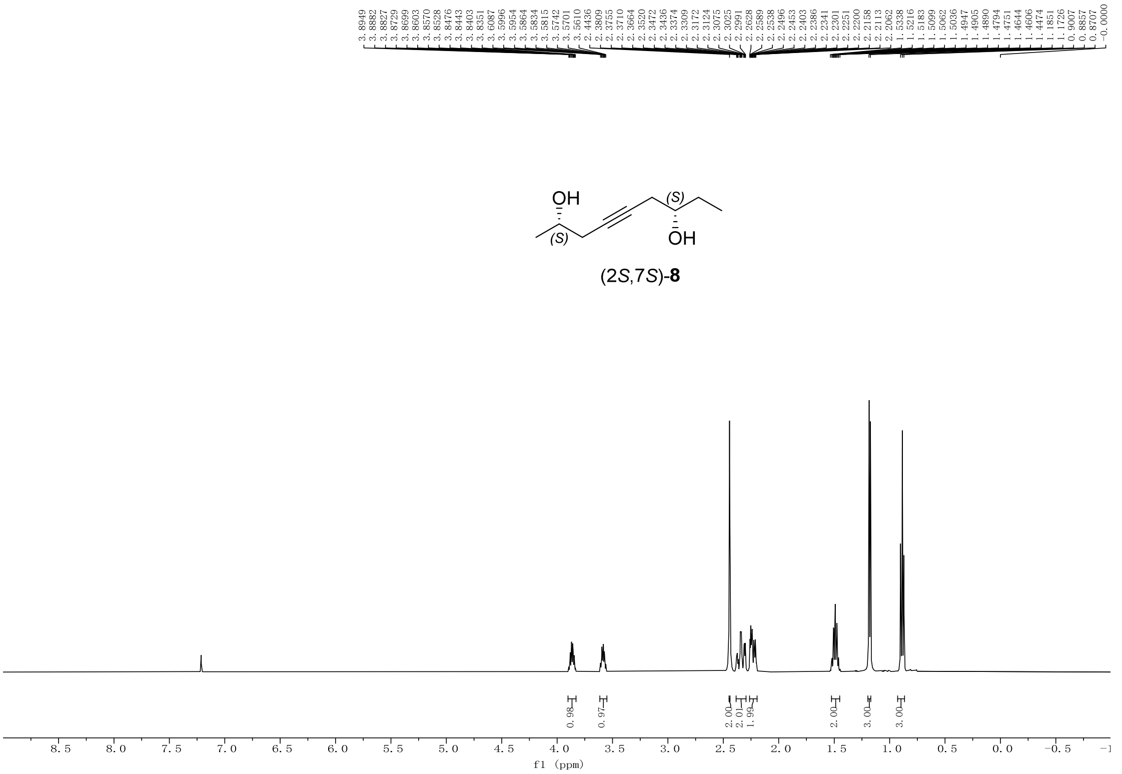

Figure S18. ^{13}C NMR Spectrum of compound (2*S*,7*S*)-**8** (125 MHz, $CDCl_3$).

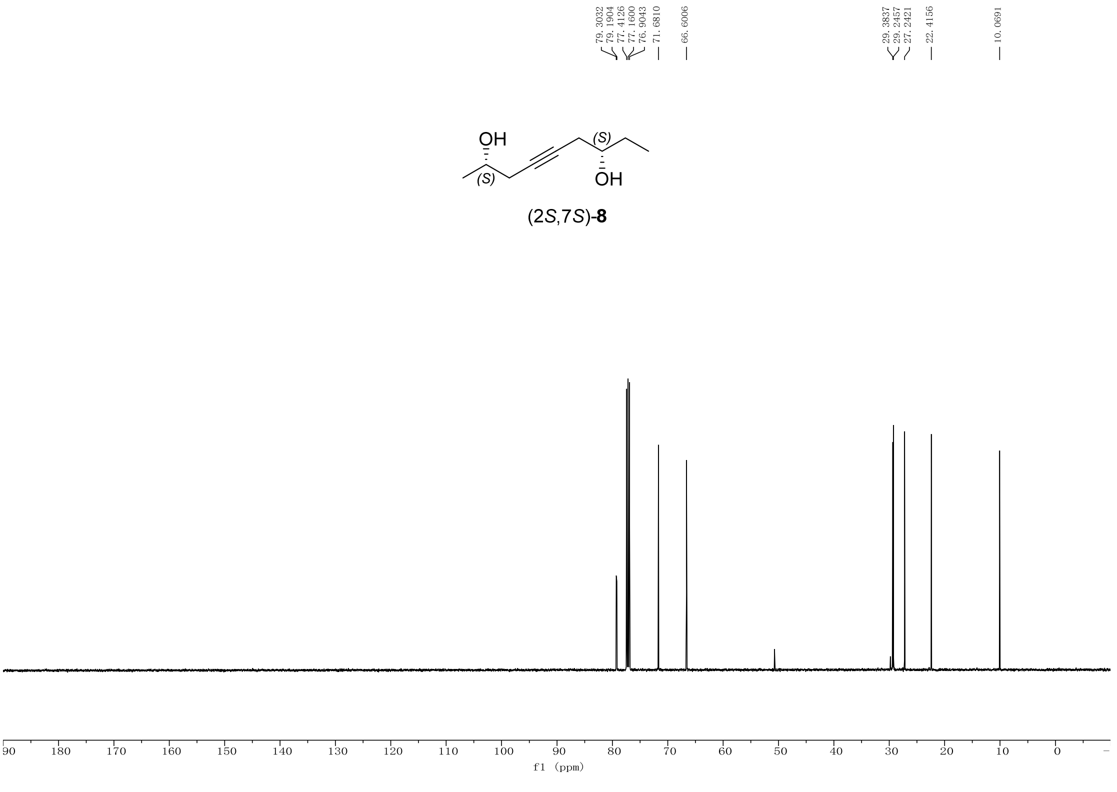

Figure S19. ¹H NMR Spectrum of compound (2*R*,7*S*)-**8** (500 MHz, CDCl₃).

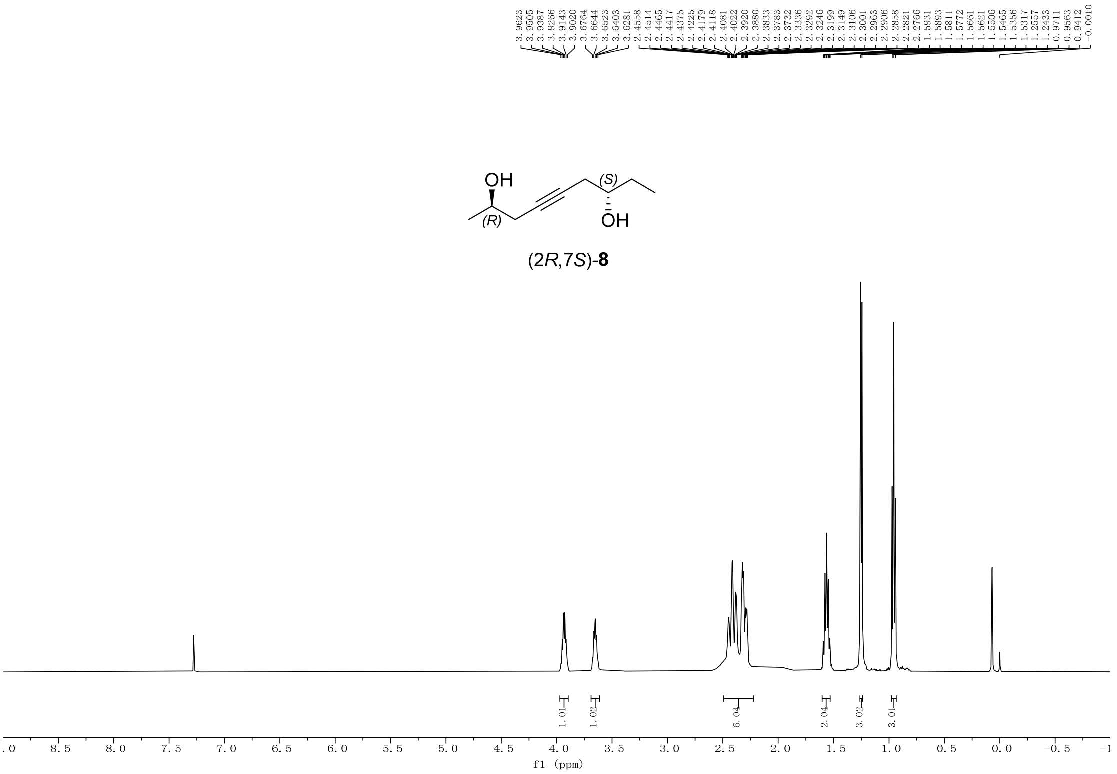

Figure S20. ¹³C NMR Spectrum of compound (2*R*,7*S*)-**8** (125 MHz, CDCl₃).

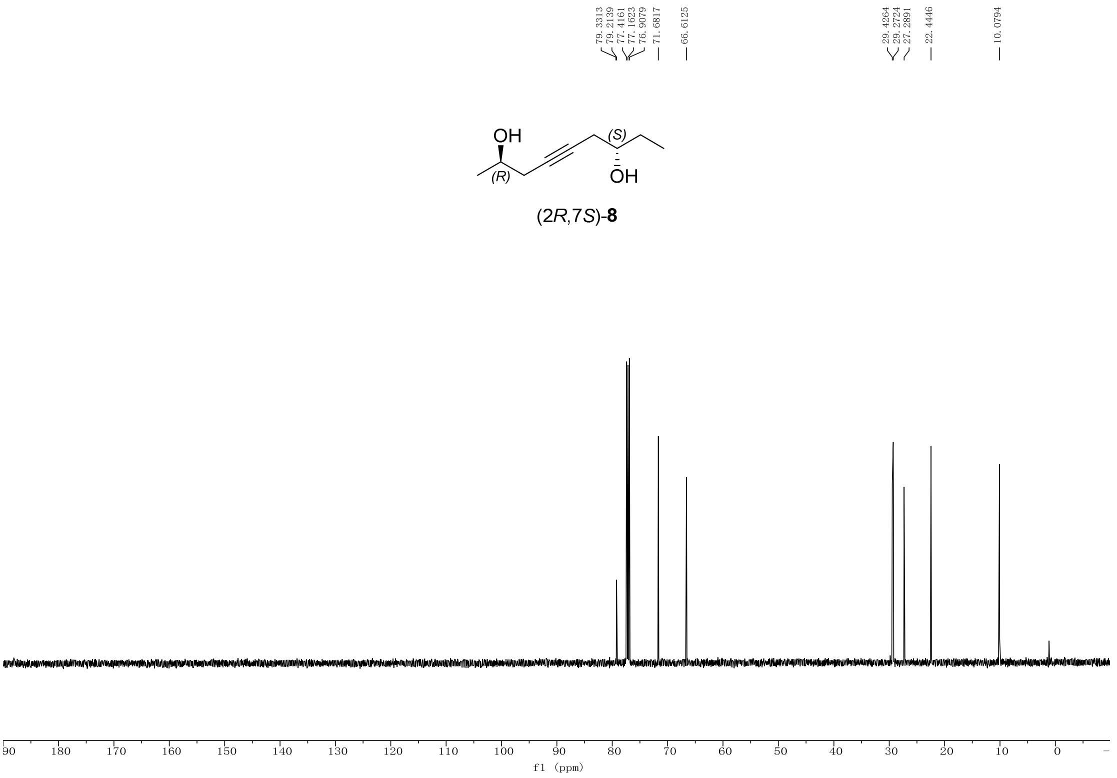

Figure S21. ^{1}H NMR Spectrum of compound (2*S*,7*R*)-**8** (500 MHz, $CDCl_3$).

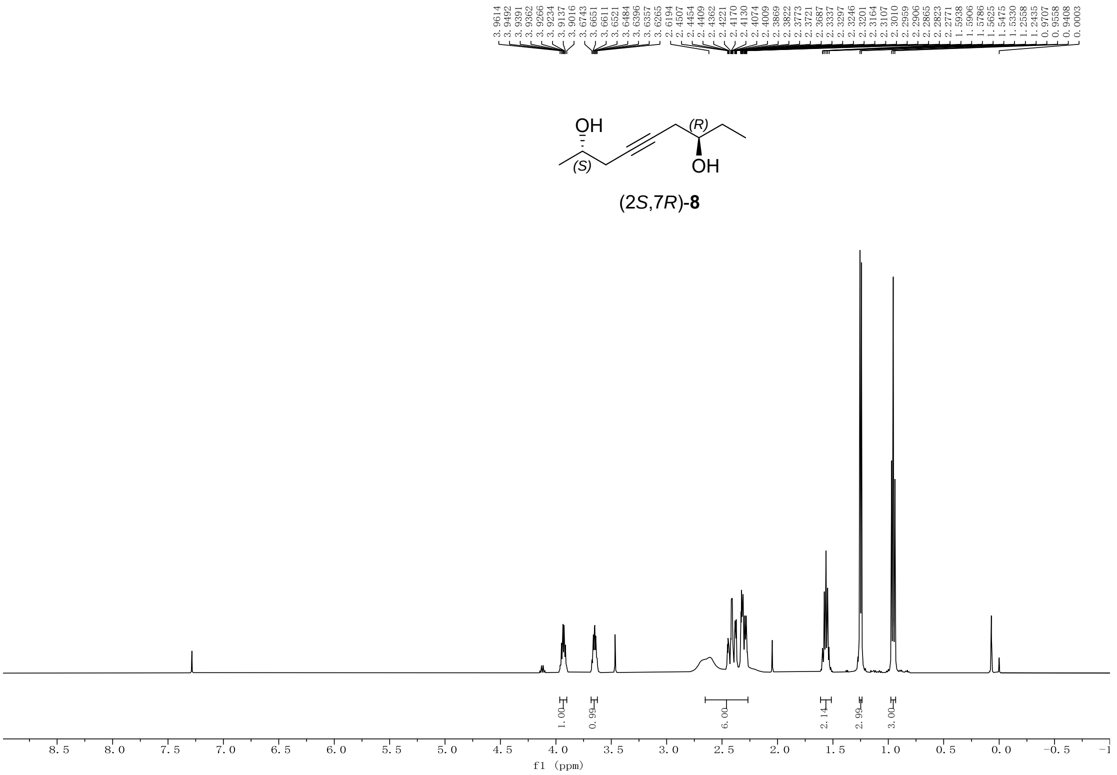

Figure S22. ^{13}C NMR Spectrum of compound (2*S*,7*R*)-**8** (125 MHz, $CDCl_3$).

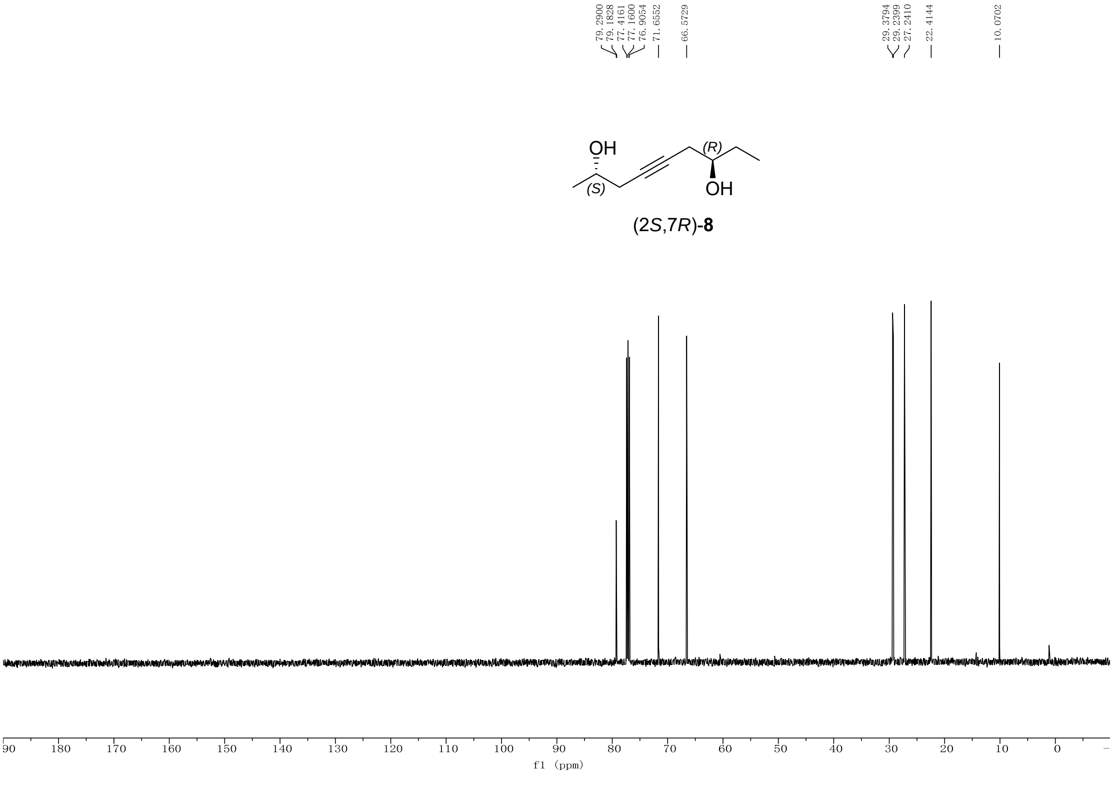

Figure S23. ^{1}H NMR Spectrum of compound (2*R*,7*R*)-**8** (500 MHz, $CDCl_3$).

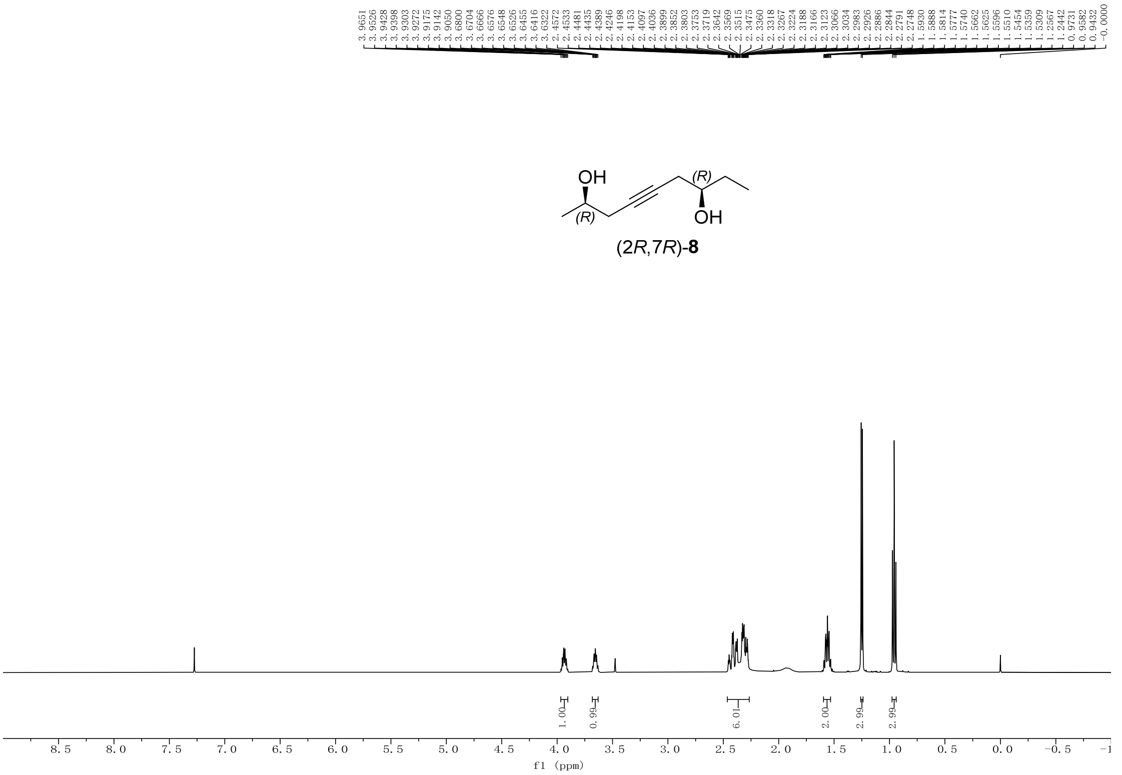

Figure S24. ^{13}C NMR Spectrum of compound (2*R*,7*R*)-**8** (125 MHz, $CDCl_3$).

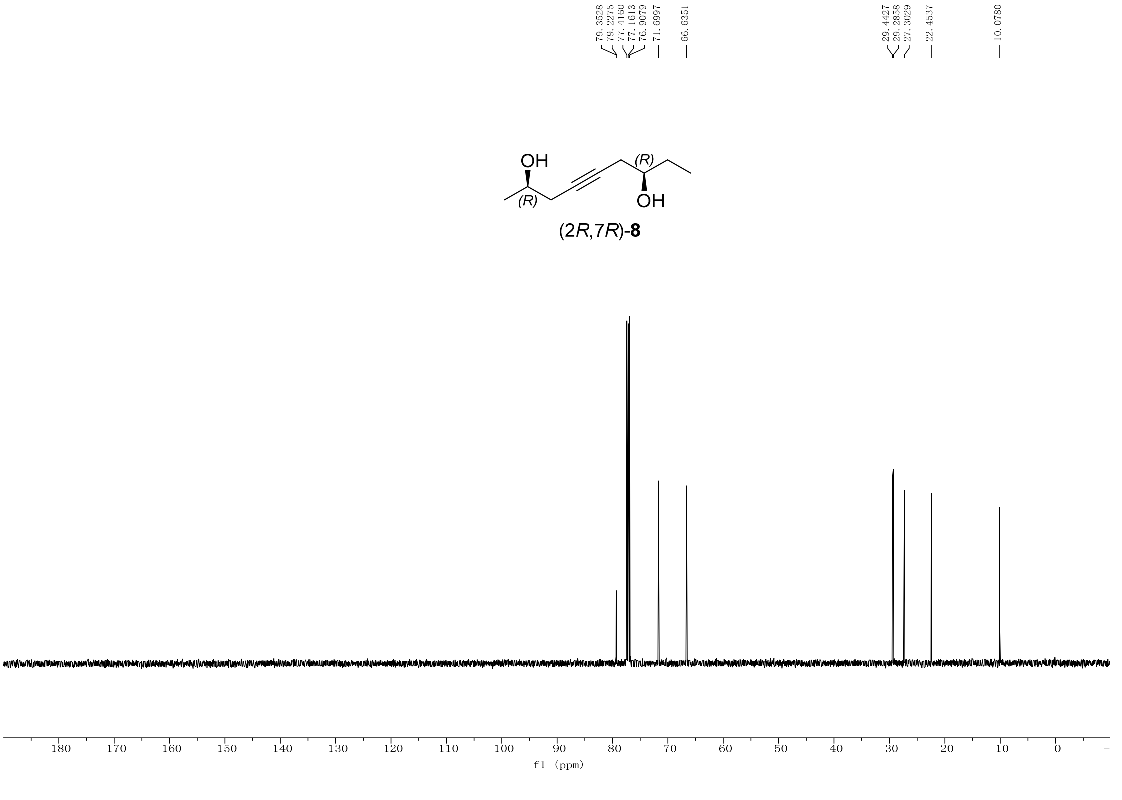

Figure S25. ^{1}H NMR Spectrum of compound (2*S*,7*S*)-**9** (500 MHz, $CDCl_3$).

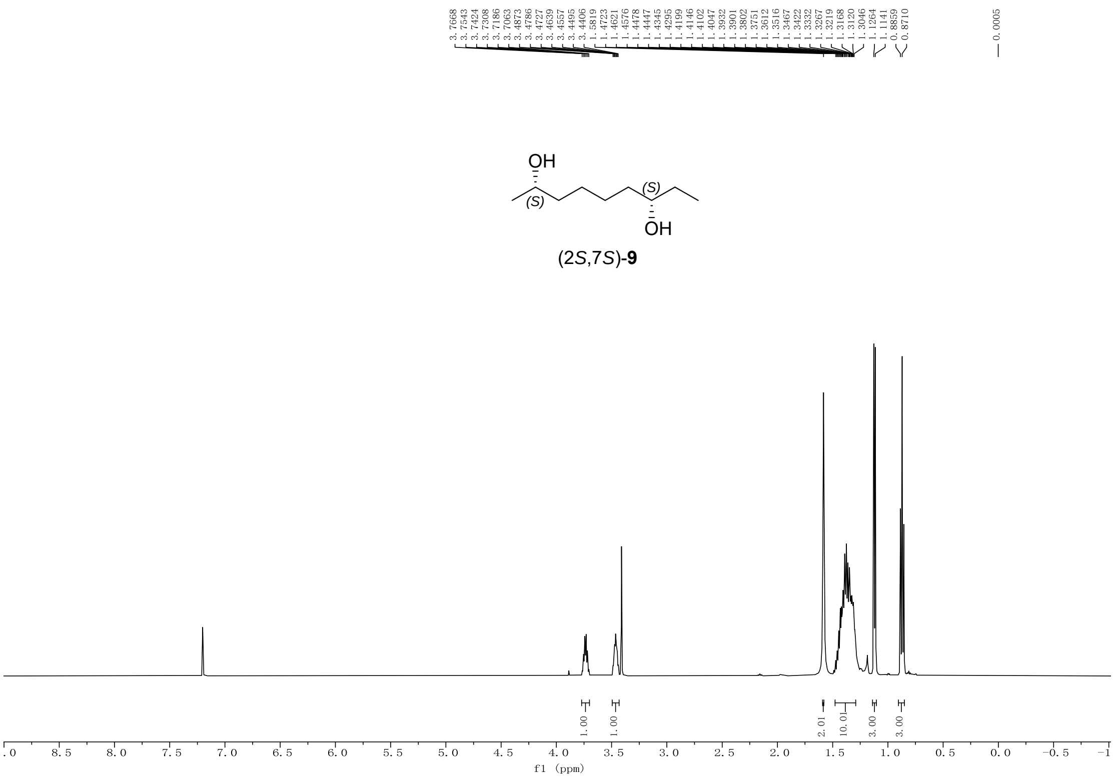

Figure S26. ^{13}C NMR Spectrum of compound (2*S*,7*S*)-**9** (125 MHz, $CDCl_3$).

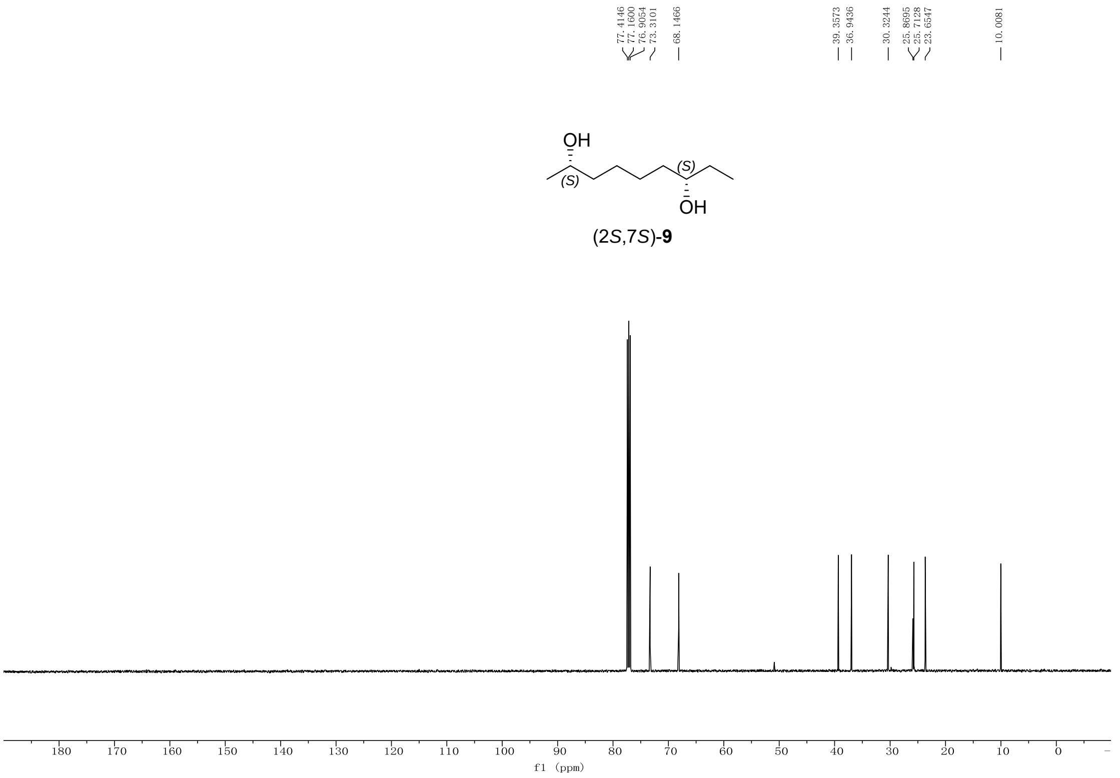

Figure S27. ^{1}H NMR Spectrum of compound (2*R*,7*S*)-**9** (500 MHz, $CDCl_3$).

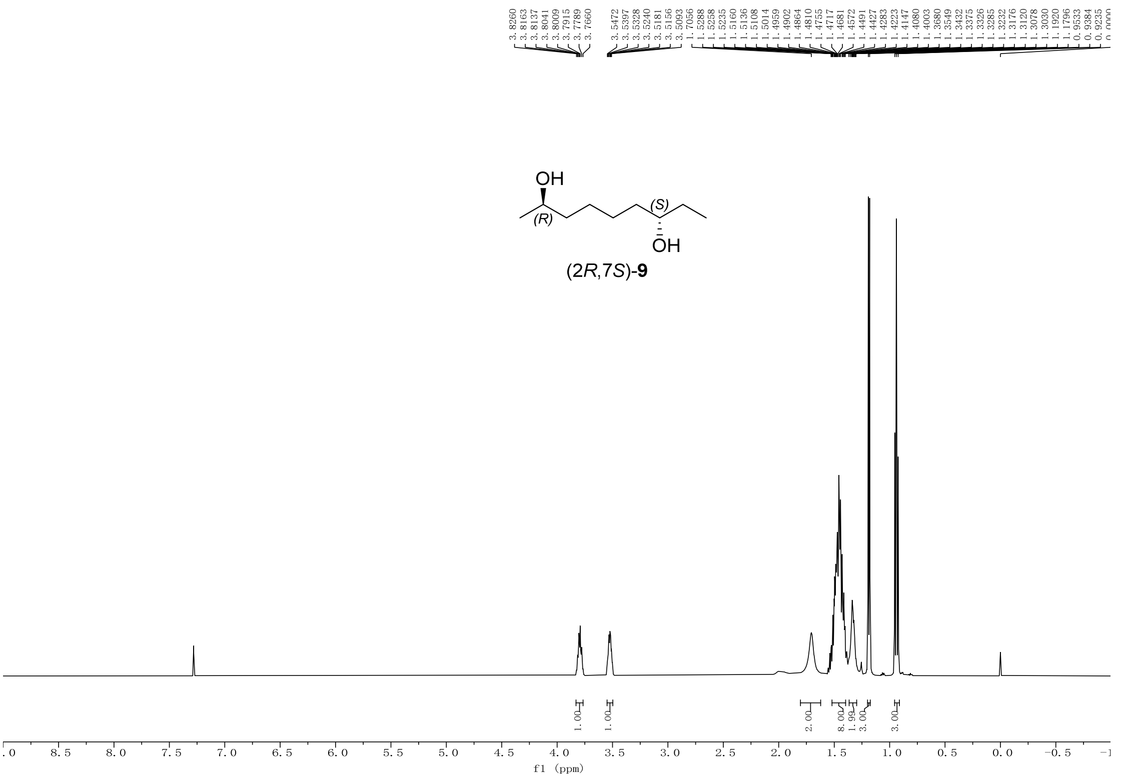

Figure S28. ^{13}C NMR Spectrum of compound (2*R*,7*S*)-**9** (125 MHz, $CDCl_3$).

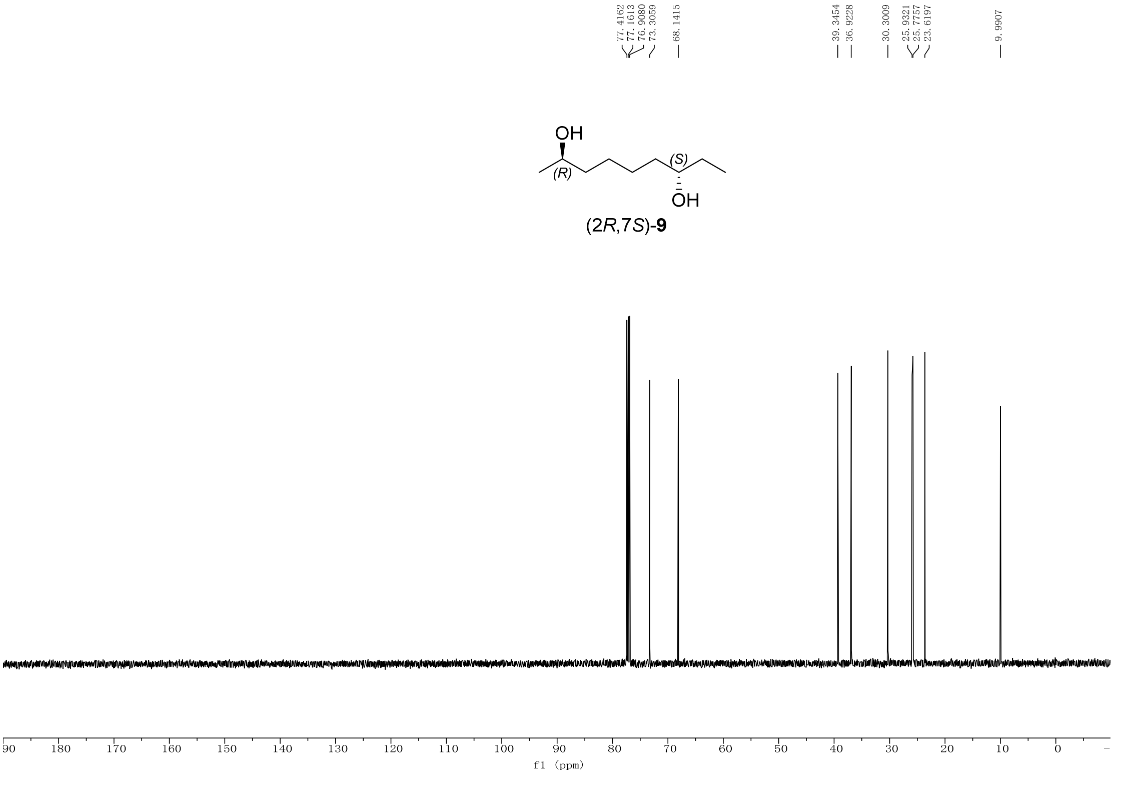

Figure S29. ^{1}H NMR Spectrum of compound (2*S*,7*R*)-**9** (500 MHz, CDCl$_3$).

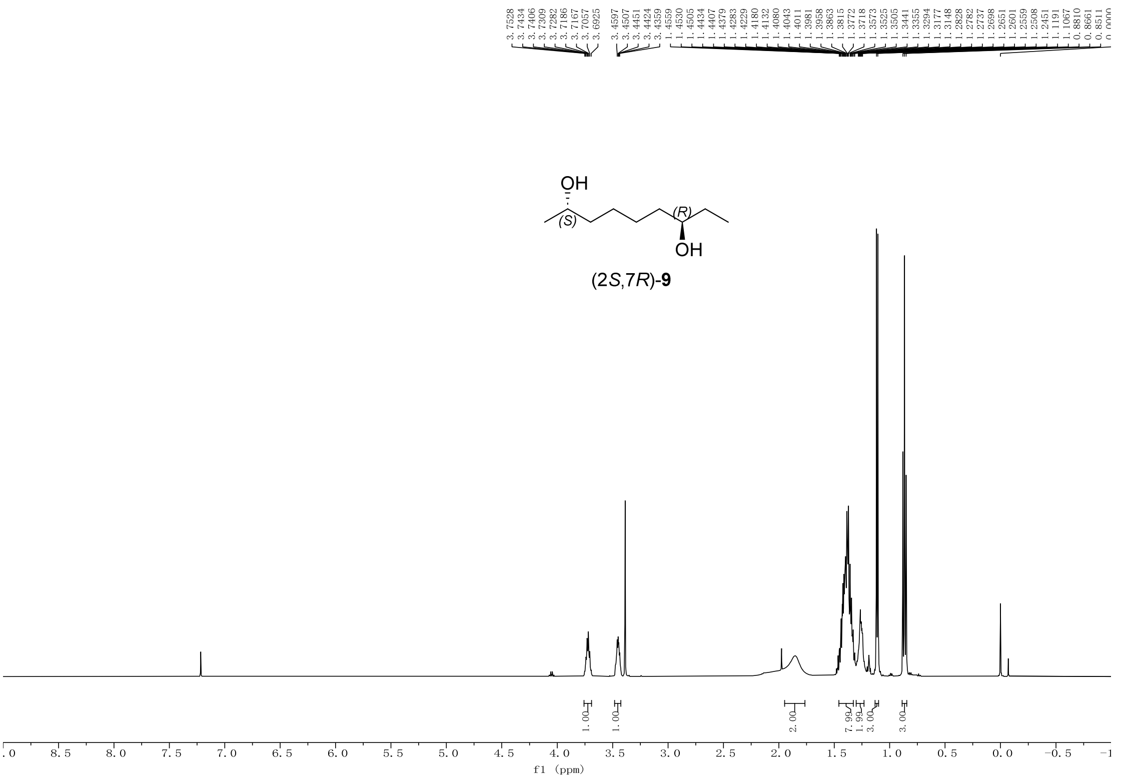

Figure S30. ^{13}C NMR Spectrum of compound (2*S*,7*R*)-**9** (125 MHz, CDCl$_3$).

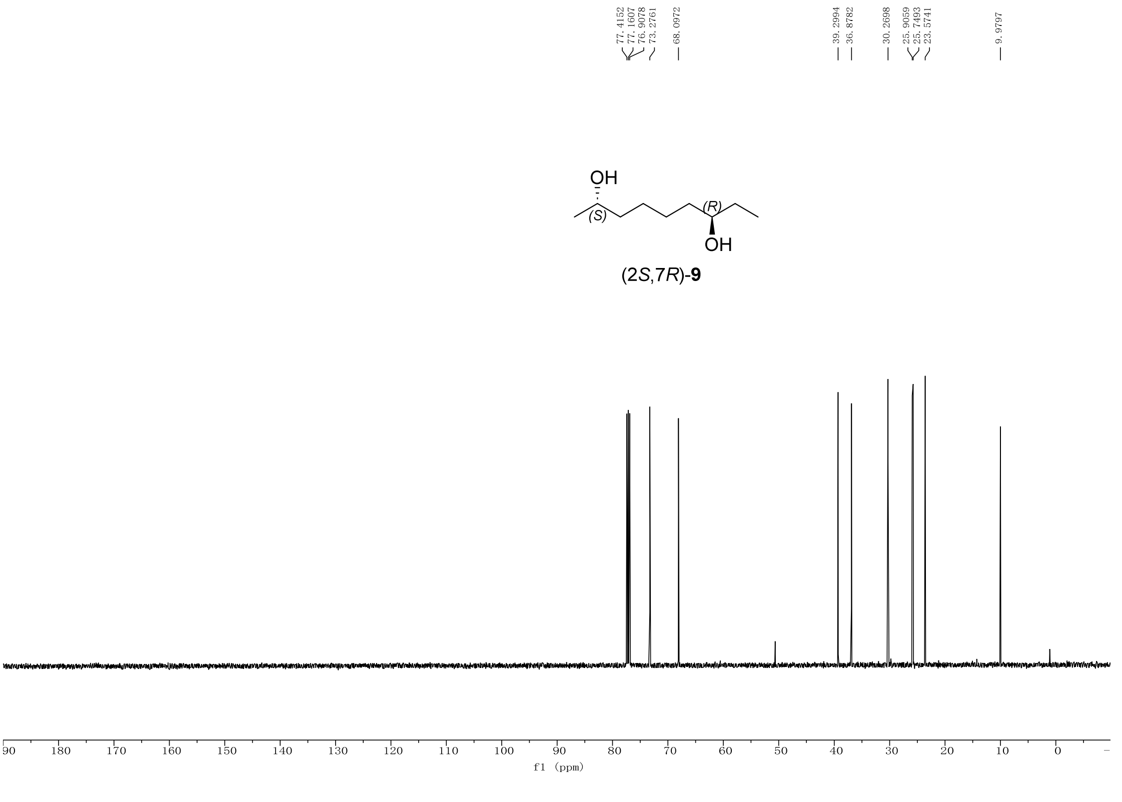

Figure S31. ^{1}H NMR Spectrum of compound (2*R*,7*R*)-**9** (500 MHz, CDCl$_3$).

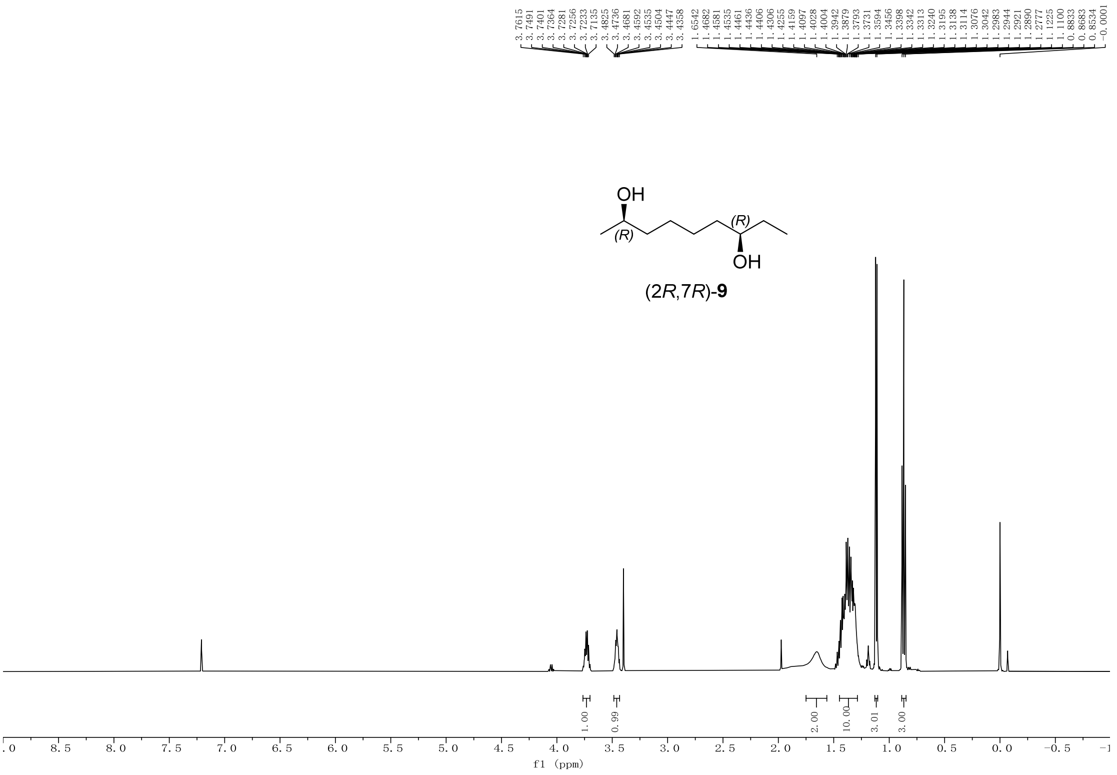

Figure S32. ^{13}C NMR Spectrum of compound (2*R*,7*R*)-**9** (125 MHz, CDCl$_3$).

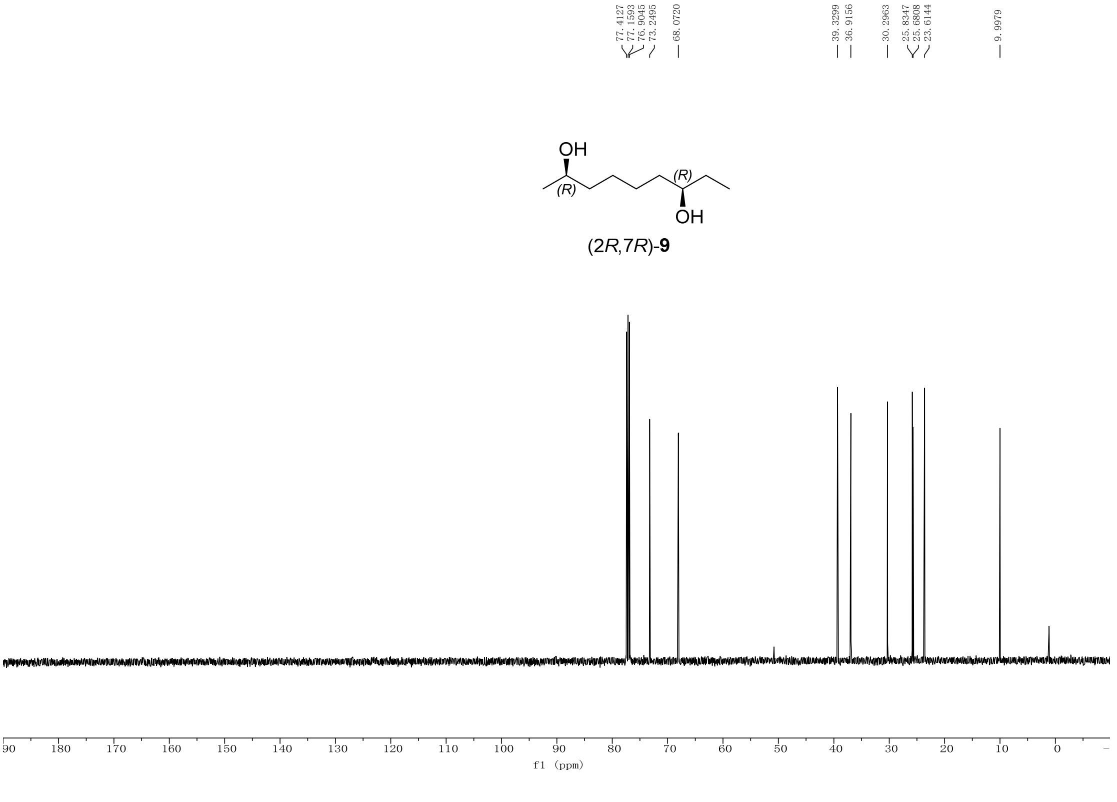

Figure S33. ^{1}H NMR Spectrum of compound (2*S*,7*S*)-**1** (500 MHz, $CDCl_3$).

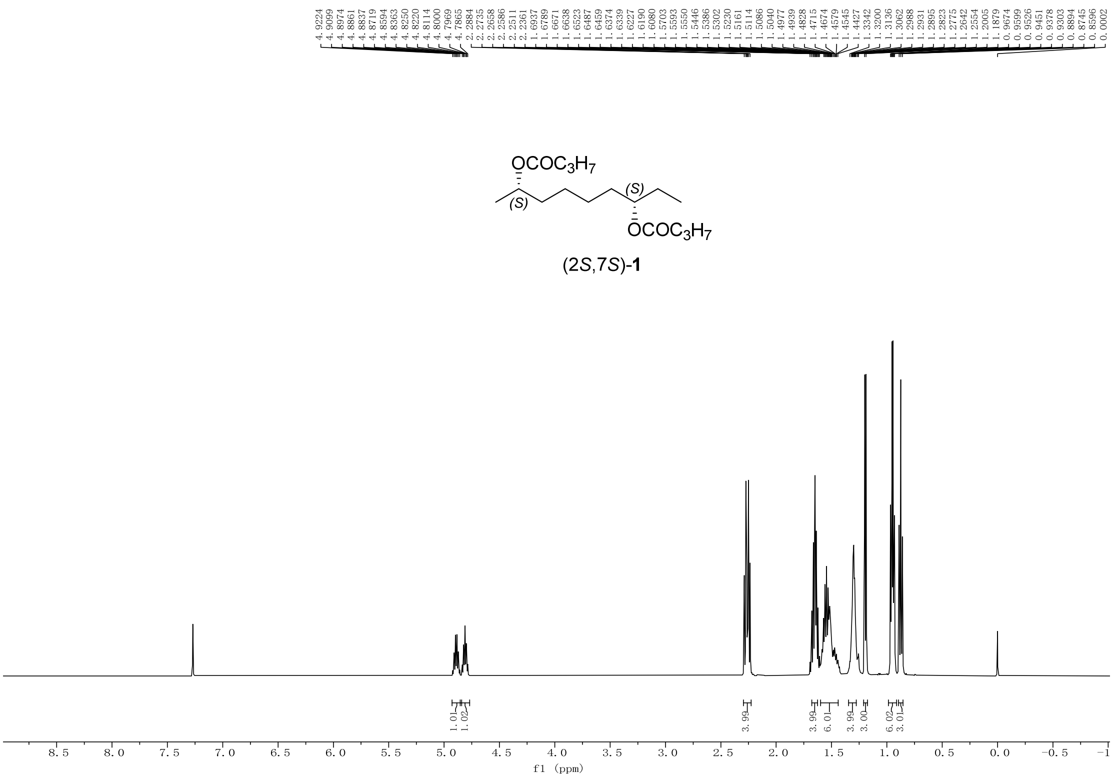

Figure S34. ^{13}C NMR Spectrum of compound (2*S*,7*S*)-**1** (125 MHz, $CDCl_3$).

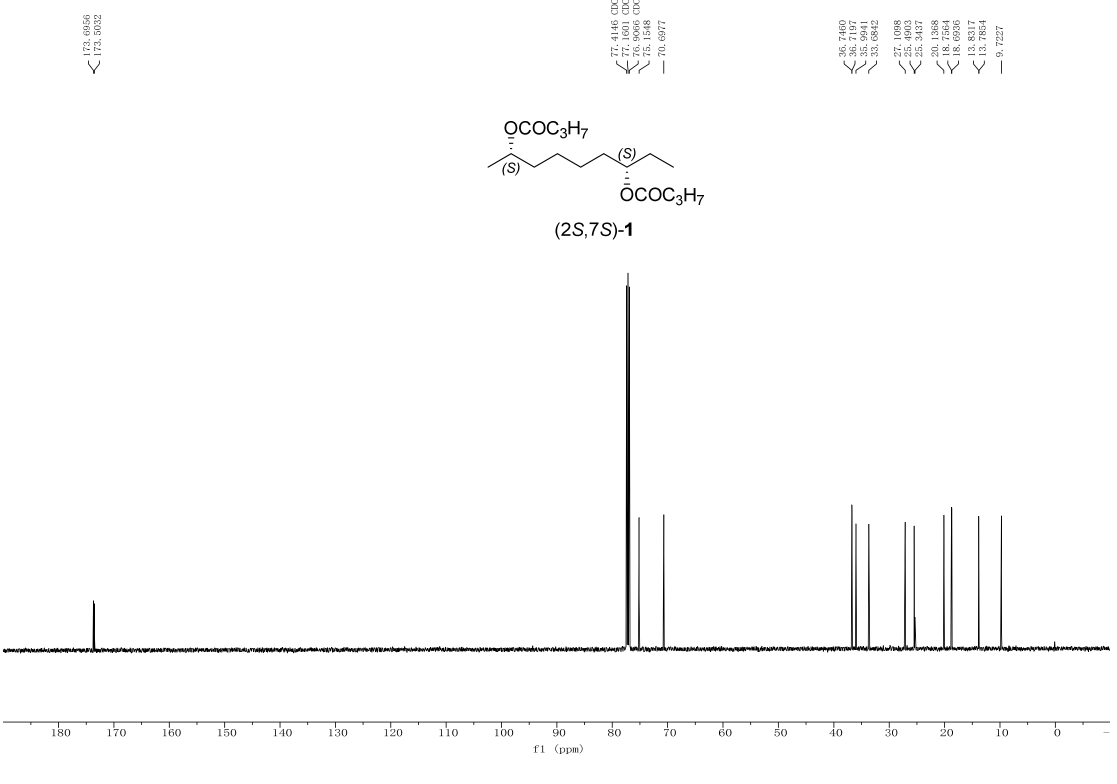

Figure S35. ^{1}H NMR Spectrum of compound (2*R*,7*S*)-**1** (500 MHz, $CDCl_3$).

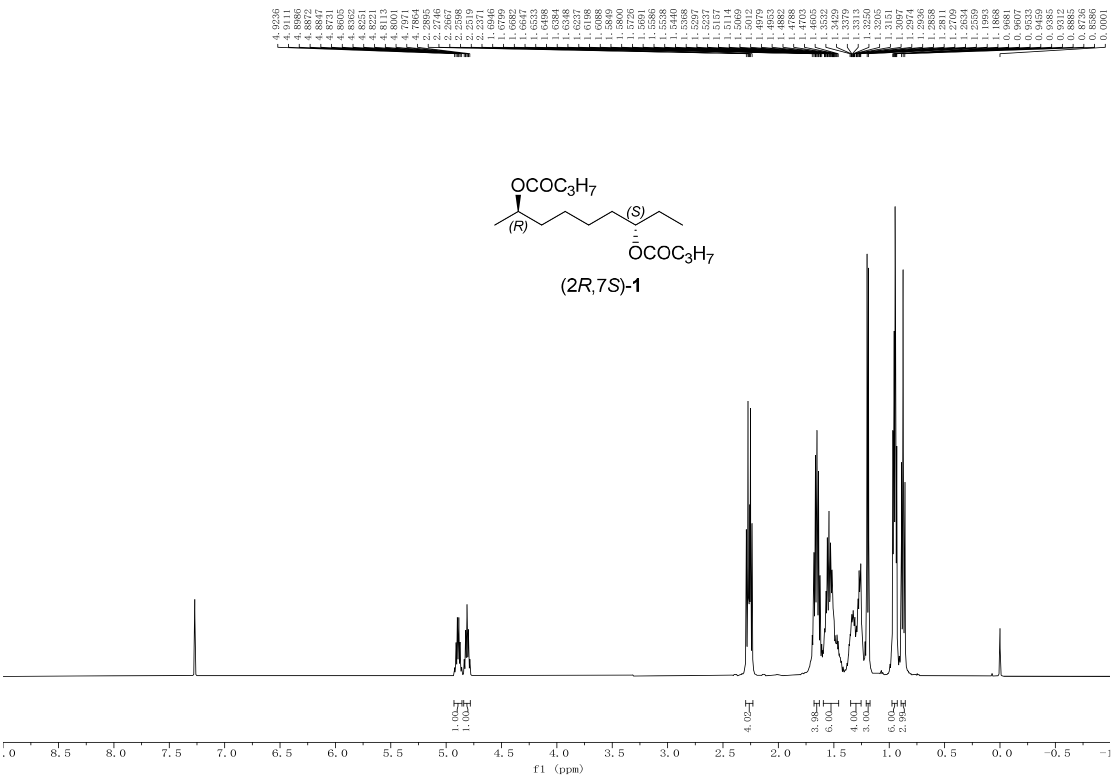

Figure S36. ^{13}C NMR Spectrum of compound (2*R*,7*S*)-**1** (125 MHz, $CDCl_3$).

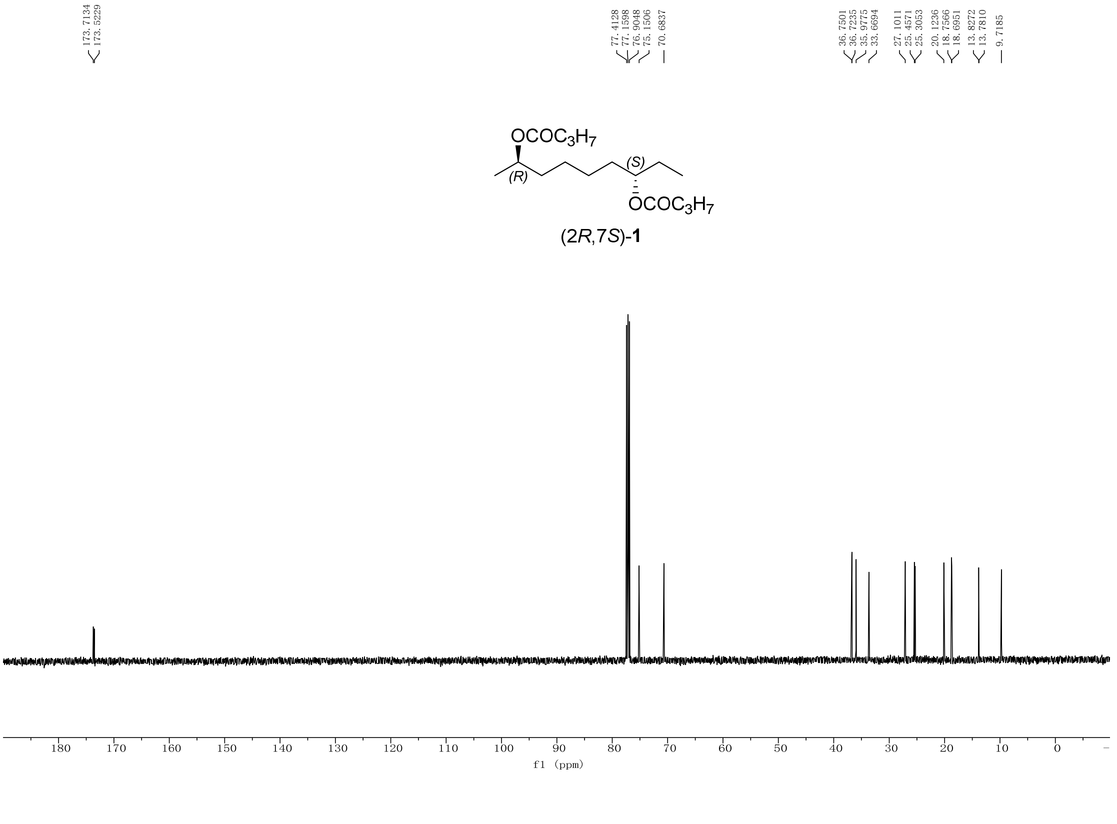

Figure S37. ^{1}H NMR Spectrum of compound (2*S*,7*R*)-**1** (500 MHz, $CDCl_3$).

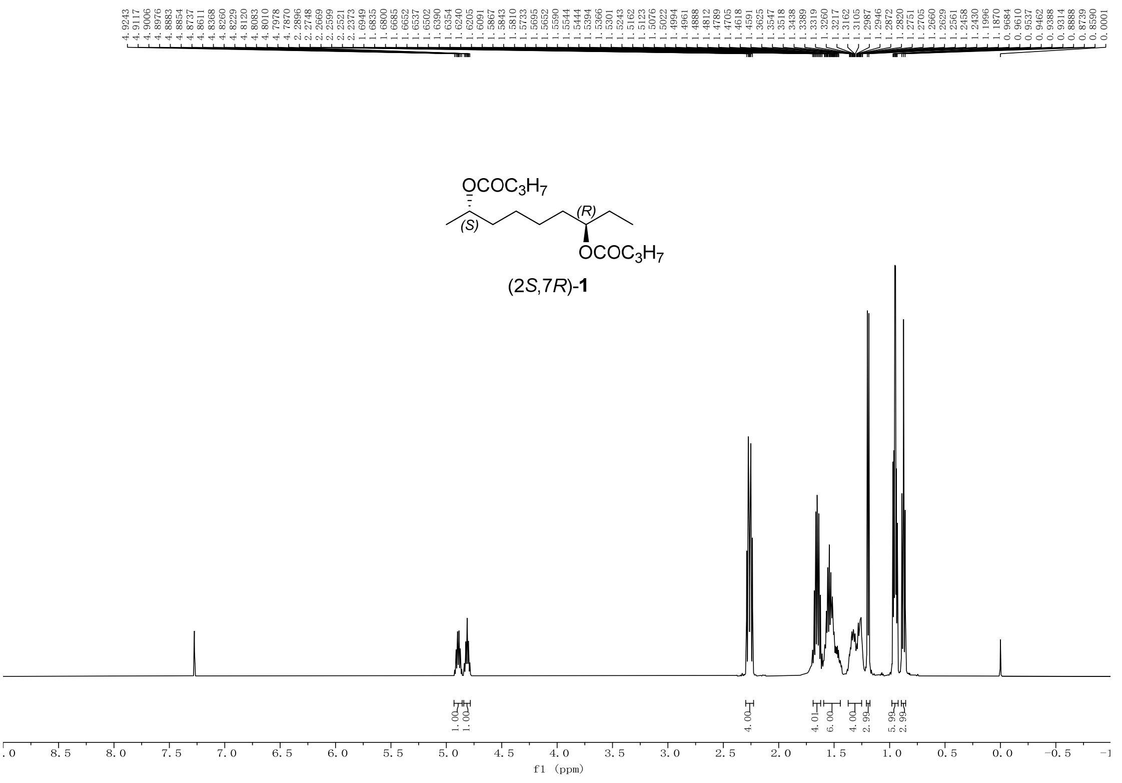

Figure S38. ^{13}C NMR Spectrum of compound (2*S*,7*R*)-**1** (125 MHz, $CDCl_3$).

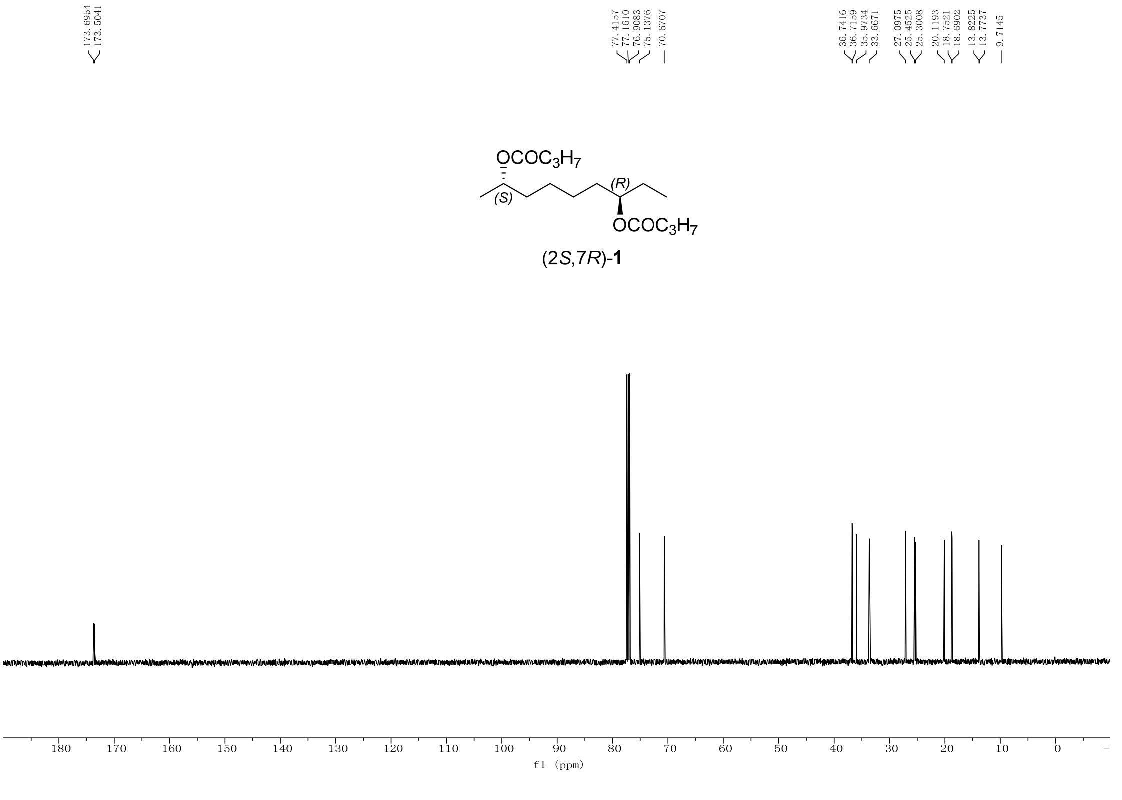

Figure S39. ^{1}H NMR Spectrum of compound (2*R*,7*R*)-**1** (500 MHz, $CDCl_3$).

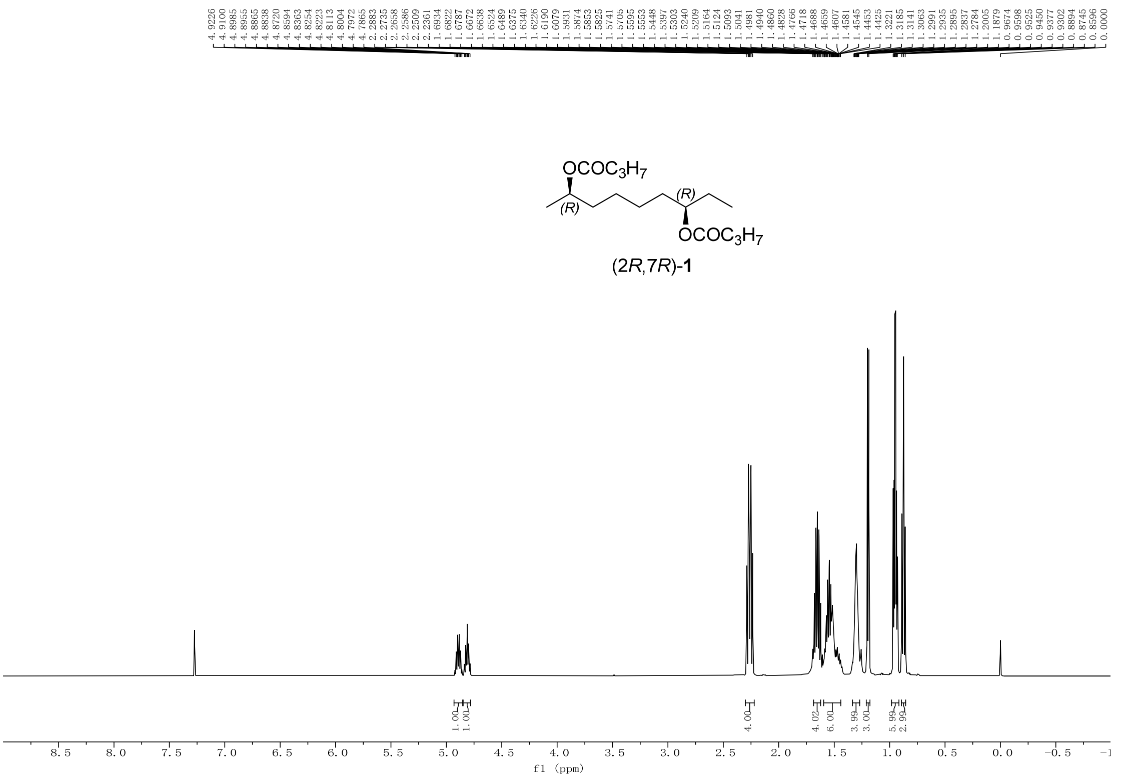

Figure S40. ^{13}C NMR Spectrum of compound (2*R*,7*R*)-**1** (125 MHz, $CDCl_3$).

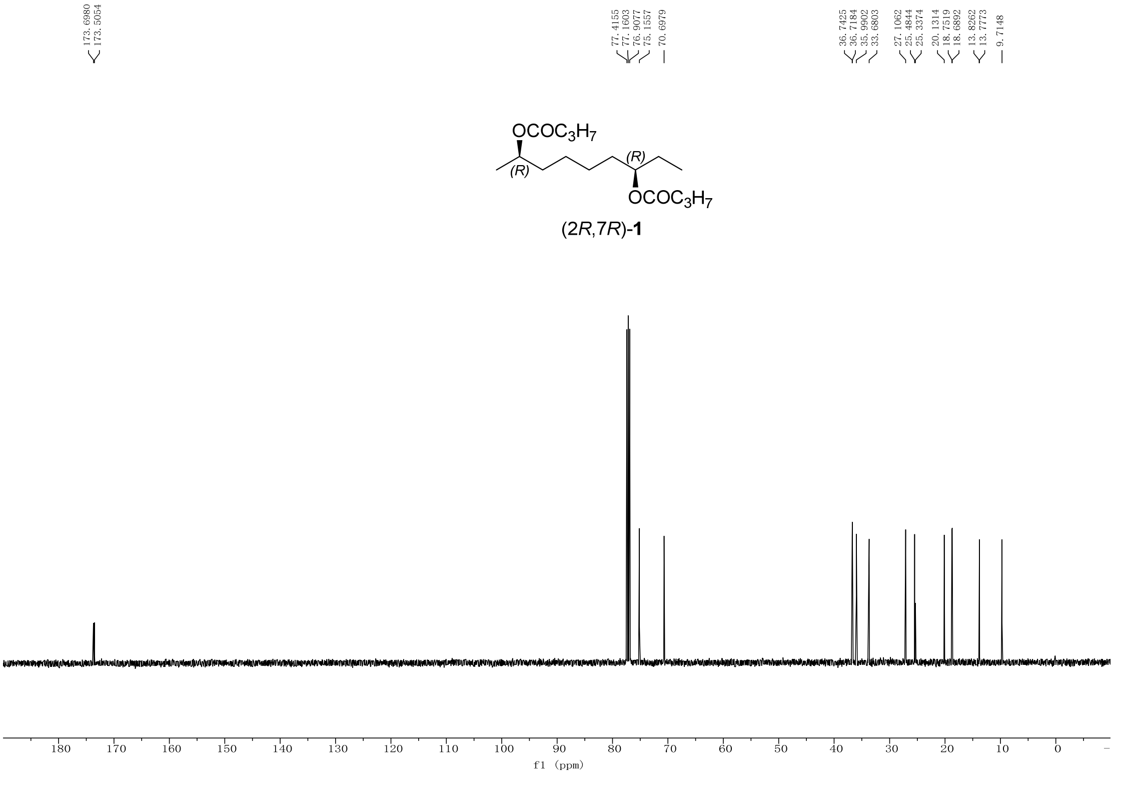

2. Comparison of NMR Data Between the Precious and Current Synthesized (2*S*,7*S*)-1

Table S1. Comparison of NMR Data between the precious [1] and current synthesized (2*S*,7*S*)-**1**.

Entry	Precious ^{1}H NMR (δ)	Current ^{1}H NMR (δ)	Precious ^{13}C NMR (δ)	Current ^{13}C NMR (δ)
1	4.84 (sex, *J* = 6.5 Hz, 1H)	4.92 – 4.86 (m, 1H)	173.4	173.7
2	4.7 6(qn, *J* = 6.5 Hz, 1H)	4.84 – 4.79 (m, 1H)	173.2	173.5
3	2.22 (t, *J* = 7.4 Hz, 2H)<br>2.20 (t, *J* = 7.4 Hz, 2H)	2.26 (dt, *J* = 11.3, 7.5 Hz, 4H)	74.9	75.2
4	1.61(sep, *J* = 7.4 Hz, 2H)<br>1.60 (sep, *J* = 7.4 Hz, 2H)	1.69 – 1.61 (m, 4H)	70.4	70.7
5	1.54 – 1.38 (m, 6H)	1.57 – 1.44 (m, 6H)	36.5	36.8
6	1.25 (m, 4H)	1.33 – 1.26 (m, 4H)	36.5	36.7
7	1.14 (d, *J* = 6.3 Hz, 3H)	1.19 (d, *J* = 6.3 Hz, 3H)	35.8	36.0
8	0.90 (t, *J* = 7.4 Hz, 3H)<br>0.89 (t, *J* = 7.4 Hz, 3H)	0.97 – 0.87 (m, 6H)	33.4	33.7
9	0.82 (t, *J* = 7.4 Hz, 3H)	0.87 (t, *J* = 7.5 Hz, 3H)	26.9	27.1
10	–	–	25.2	25.5
11	–	–	25.1	25.3
12	–	–	19.9	20.1
13	–	–	18.5	18.8
14	–	–	18.4	18.7
15	–	–	13.6	13.83
16	–	–	13.5	13.79
17	–	–	9.5	9.7

3. References

1. Hooper, A. M.; Dufour, S.; Willaert, S.; Pouvreau, S.; Pickett, J. A. Synthesis of (2*S*,7*S*)-dibutyroxynonane, the sex pheromone of the orange wheat blossom midge, *Sitodiplosis mosellana* (Gehin) (Diptera: Cecidomyiidae), by diastereoselective silicon-tethered ring-closing metathesis. *Tetrahedron Lett.* **2007**, *48*, 5991.
